# In Vivo Biosensing Using Resonance Energy Transfer

**DOI:** 10.3390/bios9020076

**Published:** 2019-06-03

**Authors:** Shashi Bhuckory, Joshua C. Kays, Allison M. Dennis

**Affiliations:** 1 Department of Biomedical Engineering, Boston University, Boston, MA 02215, USA; shashi.bhuckory@protonmail.ch (S.B.); jkays@bu.edu (J.C.K.); 2 Division of Materials Science and Engineering, Boston University, Boston, MA 02215, USA

**Keywords:** biosensor, real-time imaging, luciferase, nanoparticles, quantum dot, FRET, BRET, CRET, NIR

## Abstract

Solution-phase and intracellular biosensing has substantially enhanced our understanding of molecular processes foundational to biology and pathology. Optical methods are favored because of the low cost of probes and instrumentation. While chromatographic methods are helpful, fluorescent biosensing further increases sensitivity and can be more effective in complex media. Resonance energy transfer (RET)-based sensors have been developed to use fluorescence, bioluminescence, or chemiluminescence (FRET, BRET, or CRET, respectively) as an energy donor, yielding changes in emission spectra, lifetime, or intensity in response to a molecular or environmental change. These methods hold great promise for expanding our understanding of molecular processes not just in solution and in vitro studies, but also in vivo, generating information about complex activities in a natural, organismal setting. In this review, we focus on dyes, fluorescent proteins, and nanoparticles used as energy transfer-based optical transducers in vivo in mice; there are examples of optical sensing using FRET, BRET, and in this mammalian model system. After a description of the energy transfer mechanisms and their contribution to in vivo imaging, we give a short perspective of RET-based in vivo sensors and the importance of imaging in the infrared for reduced tissue autofluorescence and improved sensitivity.

## 1. Introduction

**Figure 1:**
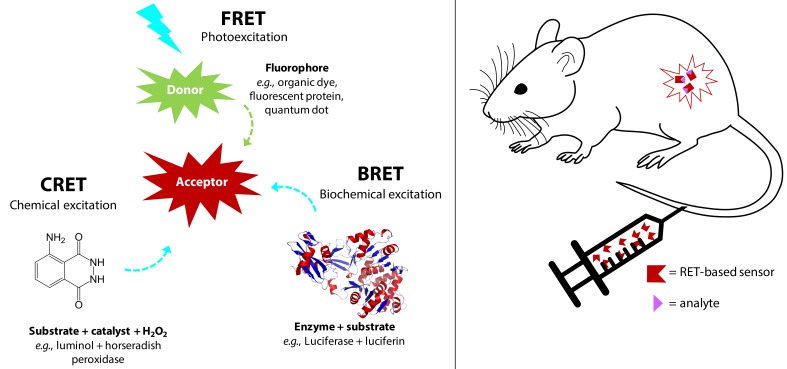
Schematic illustration of FRET, BRET and CRET-based systems, which can be injected in mice for RET-based imaging and biosensing. FRET, BRET, and CRET exploit the non-radiative energy transfer from an excited donor to an acceptor molecule in the ground-state when they are in close proximity, typically 1–10 nm. In FRET, the donor excited state occurs through external optical excitation, whereas in BRET and CRET, the donor is excited through a biochemical or chemical reaction that generates bioluminescence or chemiluminescence, respectively.

In recent years, in vivo luminescence biosensing has gained attention as a means to non-invasively probe living animals under physiological conditions with subcellular resolution [1]. The resulting images give insight into complex processes such as the evolution of disease or the impact of drug and gene therapy via in situ monitoring [2,3]. A recent review by Rong et al. describes the progress and perspectives in in vivo biosensing and mentions novel materials that could spur significant development in biomedical research and medical diagnostics [4]. Amongst other examples, Kang et al. use bioresorbable silicon for continuous monitoring of intracranial pressure and temperature in rats [5]; Unruh et al. demonstrate poly(2-hydroxyethyl methacrylate) (polyHEMA) hydrogel-based implantable sensors for the real-time measurement of glucose in pigs [6]; and Chang et al. implement nanozymes—i.e., nanoparticles with enzymatic activity mimicking natural enzymes [7]—to monitor dynamic changes in glucose concentration in the striatum (brain) of living rats [8]. We expand on this literature survey by detailing examples of resonance energy transfer-based in vivo sensors sensitive enough to probe biological tissues at the nanometer scale. In this perspective review, we focus on recent developments in utilizing resonance energy transfer (RET) processes for in vivo optical biosensing in mice. Specifically, we discuss the use of Förster resonance energy transfer (FRET), bioluminescence resonance energy transfer (BRET), and chemiluminescence resonance energy transfer (CRET) to probe biological phenomena. We begin with a brief description of the three energy transfer mechanisms and the wavelength-dependence of tissue-depth imaging, followed by examples of FRET, BRET, and CRET implementation in vivo. We conclude the review with an outlook on the future of RET-based in vivo sensors. Figure 1 represents a simplified scheme of the RET-based systems for in vivo optical sensing.

## 2. Background

### 2.1. FRET

Förster resonance energy transfer is the non-radiative energy transfer from an excited donor fluorophore to a ground-state acceptor molecule (chromophore quencher or fluorophore) in close proximity. The dipole–dipole resonance enabling FRET occurs only in the near field, a region about 1–10 nm from the donor, where the ideal dipole approximation can be applied. Muñoz-Losa et al. showed through molecular dynamics simulations that when the molecules are in an isotropic set of relative orientations, the ideal dipole approximation is valid at donor–acceptor distances as low as ca. 2 nm [9,10]. Thus, the electromagnetic interaction is a dipole–dipole interaction between the donor and acceptor, and all interactions due to higher multipoles can be ignored [9]. Dipole–dipole resonance, and thus RET, requires spectral overlap between the donor emission and the acceptor absorption. During this process, the photoluminescence (PL) intensity of the donor is quenched and its PL lifetime decreases, while the acceptor PL intensity and decay time increases, provided the acceptor is luminescent [11,12,13,14]. Energy transfer can occur between two of the same fluorophore in homogenous FRET, or homoFRET, which manifests itself as a decrease in the overall intensity of the fluorophore ensemble (i.e., self-quenching) and a red-shifting of the emission peak [15,16]. Energy transfer between two different fluorophores or a fluorophore-quencher pair is heterogeneous FRET. HeteroFRET can occur between two optically active species of diverse types, such as two organic dyes, an organic dye and organic quencher (i.e., non-fluorescent chromophore), a fluorescent semiconductor quantum dot and gold nanoparticle quencher, two fluorescent proteins, etc. [16].

According to Förster theory, we can write the FRET transfer rate, $\kappa_{FRET}$, as a function of the donor–acceptor distance [11]

(1) $$\kappa_{FRET}=\;\tau_{D}^{-1}{\left(\frac{R_{0}}{r}\right)}^{6},$$

where $\tau_{D}$ is the excited-state lifetime of the donor absent the acceptor, $R_{0}$ is the Förster distance, and r is the distance (nm) between the donor and acceptor.

The Förster distance, $R_{0}$, is the characteristic distance for a specific donor–acceptor FRET pair, at which FRET efficiency, $E_{FRET}$, is 50%, assuming $\kappa_{FRET}$ and all other decay rates are in equilibrium. FRET-pairs with large $R_{0}$ yield higher FRET efficiencies than FRET pairs with small $R_{0}$ under the same experimental conditions. $R_{0}$ can be calculated using Equation (2): [11]

(2) $$R_{0}={\left(\frac{9\left(ln10\right)\;\kappa^{2}\;\Phi_{D}}{128\;\pi^{5}\;N_{A}\;n^{4}\;}\;J\right)}^{\frac{1}{6}},$$

where $\kappa^{2}$ is a factor describing the relative orientation of the donor and acceptor transition dipoles; for a randomly oriented system, $\kappa^{2}$ is approximated as 2/3. $\Phi_{D}$ is the donor quantum yield in the absence of energy transfer. $N_{A}$ is Avogadro’s number, and n is the refractive index of the medium. J is the spectral overlap between the donor emission and acceptor absorption spectra, which describes the degree of resonance. Equation (2) can be rewritten in a simpler form to express $R_{0}$ in nm, provided J is calculated in M^−1^ cm^−1^ nm^4^, as [11]

(3) $$R_{0}=0.02108{\left(\kappa^{2}\;\Phi_{D}\;n^{-4}J\right)}^{\frac{1}{6}}.$$

Spectral overlap of the donor emission and acceptor absorption profiles is determined using the overlap integral function: [11]

(4) $$J=\int {\bar{I}}_{D}\;\varepsilon_{A}\;\lambda^{4}\;d\lambda .$$

In Equation (4), ${\bar{I}}_{D}$ represents the area normalized emission profile of the donor and is dimensionless. $\varepsilon_{A}$ is the molar extinction coefficient spectrum of the acceptor, and λ is the wavelength range of the spectral overlap in nm.

Another result of the Förster formalism is FRET efficiency, denoted here as $E_{FRET}$. FRET efficiency is defined as the fraction of energy transfer occurring per donor excitation (Equation (5)).

(5) $$E_{FRET}=\frac{\kappa_{FRET}}{\tau_{D}^{-1}+\;\kappa_{FRET}}=\frac{R_{0}^{6}}{R_{0}^{6}\;+\;r^{6}}=\frac{n_{A}R_{0}^{6}}{n_{A}R_{0}^{6}+r^{6}}$$

By substituting Equation (1) for the $\kappa_{FRET}$ term in Equation (5), we see that $E_{FRET}$ is inversely proportional to *r*^6^. When many acceptors are clustered around a single donor, we must account for this multivalency, where $n_{A}$ is the average number of acceptor molecules per donor molecule. In such FRET systems, $E_{FRET}$ increases with $n_{A}$. The severe distance dependence of $E_{FRET}$ renders energy transfer effective between 0.5$R_{0}$ and 2$R_{0}$, which corresponds to biologically-relevant scales for $R_{0}$ between 4–10 nm, as is typical for energy transfer using dyes, fluorescent proteins, quantum dots (QDs), and/or lanthanide complexes [11,15,16,17].

FRET efficiency can also be calculated directly from luminescence (Equation (6)) of the FRET donor by measuring the quantum yield, decay time, or intensity of the donor alone and in the presence of the acceptor.

(6) $$E_{FRET}=1-\;\frac{\Phi_{DA}}{\Phi_{D}}=1-\;\frac{I_{DA}}{I_{D}}=1-\;\frac{\tau_{DA}}{\tau_{D}}$$

where $\Phi_{DA}$ and $\Phi_{D}$ are the donor quantum yields, $I_{DA}$ and $I_{D}$ are the fluorescence intensity of the donor, and $\tau_{DA}$ and $\tau_{D}$ are the donor photoluminescent lifetimes, each in the presence and absence of the acceptor, respectively [11].

### 2.2. BRET and CRET

Bioluminescence resonance energy transfer (BRET) is an analogue to FRET, where the donor is a bioluminescent molecule and thus does not require external photoexcitation. BRET-based energy transfer follows FRET theory and thus can similarly be used to generate a change in signal in response to a nanometer scale change in distance. Multiple advantages arise from using a sensing and imaging modality that does not require external excitation of the donor molecule: no photobleaching of the donor, minimal biological autofluorescence, higher signal-to-noise ratio, and no background from direct acceptor excitation and fluorescence [11,18,19]. Bioluminescence (BL), as indicated by its name, is the generation of luminescence in a living organism through a biochemical reaction requiring two main components: luciferase and luciferin [20]. Luciferase and luciferin are generic terms for a number of different enzymes and small organic molecules, respectively, that have been developed either through natural evolution or genetic engineering [20,21,22]. Luciferins are luminogenic substrates that produce visible light upon oxidation catalyzed by luciferase in the presence or absence of cofactors [20,21]. The emission wavelength of specific luciferase/luciferin pairs depends on several factors such as the sequence of the luciferase and the structure of the luciferin (Table 1) [18,21,23,24,25,26,27].

**Figure 2:**
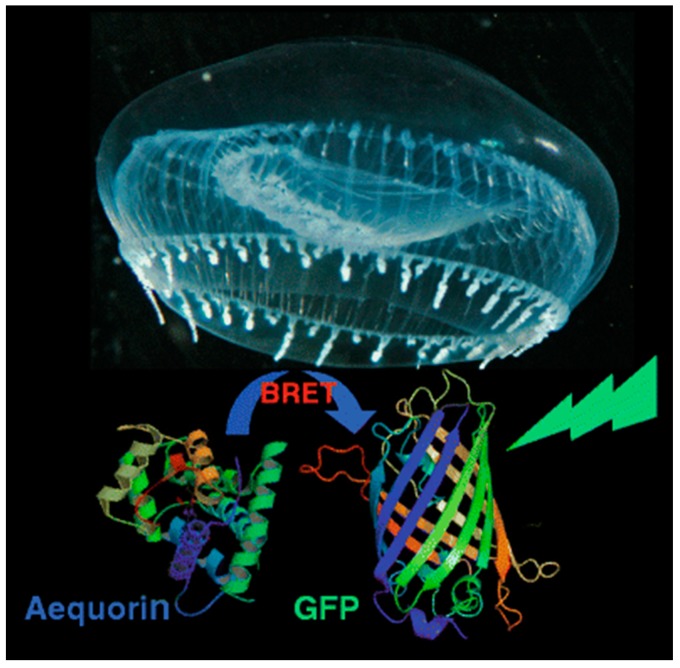
BRET in nature. Both the Ca^2+^-dependent blue-emitting protein aequorin and green fluorescent protein (GFP) are naturally co-localized in rings around the bell of the jellyfish *Aequorea victoria*. When aequorin is excited in response to a pulse of Ca^2+^ flooding the area, the resulting energy can be transferred to nearby GFP, shifting the natural luminescence of the organism from blue to green through BRET. Reproduced with permission from [32]. Copyright 2010 Springer Nature.

As an example, aequorin, a photoprotein, can catalyze the oxidation of the substrate coelenterazine (CLZN) in the presence of Ca^2+^, triggering emission at 470 nm [31]. Aequorin was discovered and originally isolated from the jellyfish *Aequorea victoria*, which is famously also the natural source of the original green fluorescent protein (GFP). Interestingly, this jellyfish provides an example of naturally occurring BRET: blue emission from aequorin is generated in response to a pulsed release of calcium ions [32]. This energy can be transferred to co-localized GFP, resulting in green fluorescent emission from a ring around the edge of the jellyfish bell (Figure 2). 

**Figure 3:**
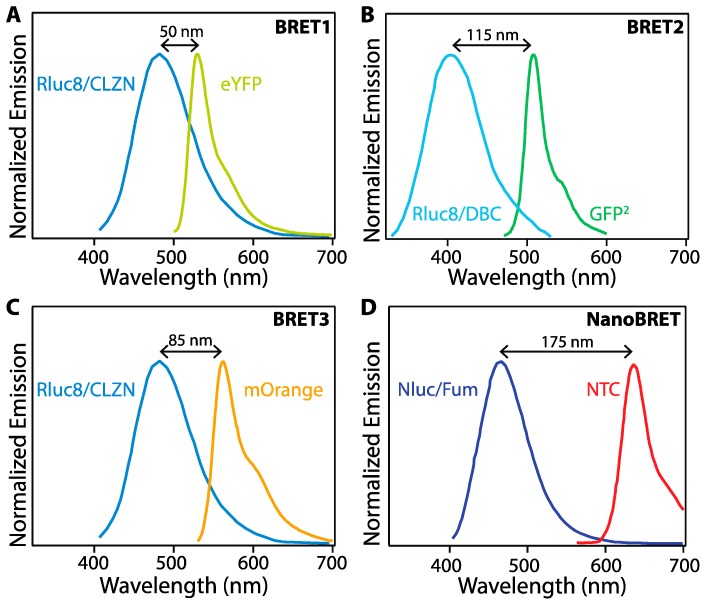
Comparison between different BRET systems. (**A**) BRET1 with Rluc8 mutant of Rluc as BRET donor and enhanced yellow fluorescent protein (eYFP) as BRET acceptor. (**B**) BRET2 with Rluc8 coupled with the substrate DeepBlueC (DBC) as the BRET donor and a green fluorescent protein variant (GFP^2^) as the BRET acceptor. (**C**) BRET3 using Rluc8/mOrange BRET-pair with coelenterazine as the substrate. (**D**) NanoBRET uses Nluc with a novel coelenterazine derivative, furimazine, and a chloroalkane derivative of nonchloro TOM (NCT) dye, which covalently binds to a HALO tag fused to Nluc. Adapted from [19,36].

The original, and still most common, BRET donors are native BL proteins such as Renilla luciferase (Rluc) or aequorin. BL generated with Rluc in the presence of its substrate coelenterazine (CLZN) and O_2_ exhibits an emission maximum at 480 nm, while using the substrate DeepBlueC (a derivative of CLZN) blue-shifts the BL emission to 395 nm, demonstrating tunability of BL based on substrate (Figure 3A,B). Oxidation of D-luciferin catalyzed by firefly luciferase (Fluc) generates an emission peak maximum between 560 and 580 nm in the presence of the luciferase cofactors ATP and Mg^2+^ [21,33]. A more efficient luciferase called NanoLuc (Nluc) was recently engineered from the deep-sea shrimp *Oplophorus gracilirostris*. BL produced with Nluc in the presence of a new substrate called furimazine emits light at 460 nm with an emission half-life of more than 2 h and demonstrates a 150-fold superior luminescence compared to Fluc or Rluc [22]. 

The emission peak wavelength and width critically impact the effectiveness of various BL proteins as BRET donors. An early BRET pair, denoted BRET1, combined the Rluc8 mutant of Rluc and CLZN as a BRET donor with enhanced yellow fluorescent protein (eYFP) as the BRET acceptor. While energy transfer was detectable in vitro, the wide emission peak of Rluc/CLZN overlaps significantly with the emission of eYFP (Figure 3A), leading to spectral crosstalk in the acceptor emission channel and a low signal-to-noise ratio, negatively impacting assay sensitivity. BRET2 uses Rluc8 as BRET donor and a green fluorescent protein variant (GFP^2^) as BRET acceptor with DeepBlueC (DBC) as a substrate to improve the spectral separation between the donor and acceptor emission. While spectrally improved, this system exhibits suboptimal performance due to low luminescence quantum yield and stability [19]. BRET3 using Rluc8/mOrange BRET-pair with coelenterazine as the substrate exhibits red-shift light emission for improved imaging in animal models and several-fold better light emission compared to BRET1 and BRET2 [34]. Numerous additional BRET pairs have been demonstrated to continue to improve the photophysical properties of the protein fusions for enhanced sensitivity and wavelength tuning [35].

In addition to fluorescent proteins (FPs), BRET acceptors include organic dyes and nanoparticles (NPs) with high brightness such as semiconductor quantum dots (QDs) [25,26,27,33]. In one example, BRET between Nluc with a novel coelenterazine derivative, furimazine, and a chloroalkane derivative of nonchloro TOM (NCT) dye, exhibits red-shifted emission with virtually no optical crosstalk between Nluc/furimazine BL emission and NCT emission spectra, thereby achieving a high signal-to-noise ratio. NanoBRET shows enhanced luminescence intensity as compared to the other BRET systems shown in Figure 3, but requires the addition of an organic dye, whereas the others are expressed as fusion proteins [36]. Novel furimazine analogues paired with Nluc can red-shift BL from 460 nm to 598 nm, making them efficient BRET donors to red fluorophores, including red-fluorescent proteins [28,36].

Chemiluminescence (CL) is a broader descriptor that encompasses bioluminescence as well as synthetically derived oxidase-catalyzed luminescence. Chemiluminescence resonance energy transfer (CRET) similarly follows FRET theory with the donor molecule excited through a chemical reaction [11]. CL substrates are synthetic compounds such as luminol (which can enter cells and tissues easily [37]) and its derivatives like 1,2-dioxetanes or acridinium esters [38]. CL is emitted upon the de-excitation of the excited intermediate molecule 3-aminophthalate, formed when luminol is oxidized by an oxidant such as hydrogen peroxide (H_2_O_2_) with help from a catalyst like a metal ion or a redox enzyme [29]. Horseradish peroxidase (HRP) has been widely used to catalyze luminol (and its derivatives) to generate CL at 425 nm in the presence of H_2_O_2_. Oxidization of acridan substrates by H_2_O_2_ and HRP emit light at 530 nm. Another substrate produces chemiluminescence at 480 nm and 530 nm by the enzymatic oxidation of adamantyl-1,2-dioxetane by alkaline phosphatase [18]. CRET works with similar acceptors as BRET and FRET.

### 2.3. Tissue Depth Imaging

**Figure 4:**
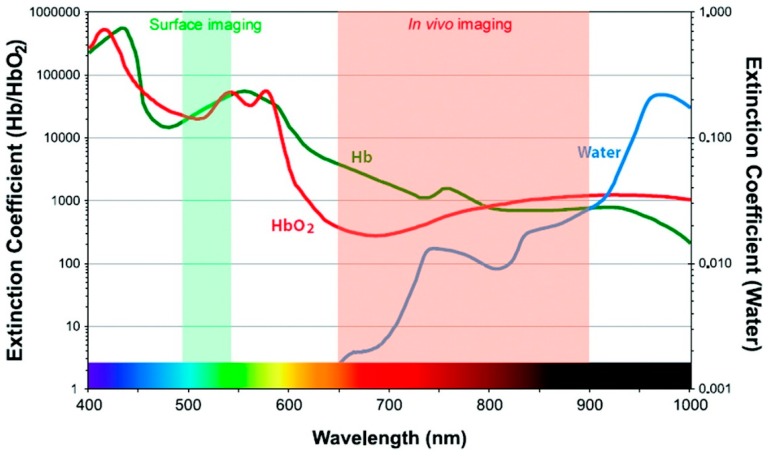
Extinction coefficient values of water and oxy- and deoxyhemoglobin across wavelengths of light used for imaging. Reprinted with permission from [44]. Copyright 2010, American Chemical Society.

In addition to developing resonance energy transfer constructs, effective use of RET-based sensing in vivo requires that constraints on luminescence imaging in animals be taken into consideration. Fluorescent tags can be readily bioconjugated to biomarker-specific recognition moieties, allowing selective targeting and visualization in vivo [39]. The fluorescent probes used in clinical diagnostics are typically organic molecular dyes generally requiring ultraviolet (UV) or visible (VIS) light excitation for the emission of visible light [40]. UV light excitation has been reported to induce phototoxic effects in biological tissues, autofluorescence resulting in low signal-to-noise ratio, and photobleaching of organic dyes [41]. The acquisition of fluorescence images takes only seconds to minutes, but the signal is vulnerable to attenuation with increased tissue depth [2]. UV and visible light is attenuated through absorption and scattering in tissue, precluding deep tissue imaging, but several optical tissue windows in the near infrared (NIR) range enable light of those wavelengths to penetrate millimeters deep [39,41]. For example, in human skin, UV light can only shallowly penetrate the upper layer, while red light can reach ~ 6 mm below the surface of the skin [42]. While this is shallow compared to non-optical methods like positron emission tomography (PET), computed tomography (CT), X-ray, or magnetic resonance imaging (MRI), there are advantages to optical imaging that compliment other approaches, such as the use of non-ionizing radiation, high spatial resolution, and relatively low-cost instrumentation [43]. Absorption by molecules prominent in biological tissues contributes strongly to the attenuation of light in tissues (Figure 4), as does scattering. There are three major biological windows where scattering and/or absorption is minimized: NIR-I (650–950 nm), NIR-II (1000–1350 nm), and NIR-III (1550–1870 nm) [41]. While fluorescence signals in the visible region of the electromagnetic spectrum have been shown to be appropriate for superficial in vivo imaging applications, NIR light is ideally used for whole body imaging of small animals due to increased penetration depths [39].

## 3. In Vivo FRET

FRET-based biosensing has been used extensively in solution assays [45,46,47,48,49,50,51,52,53,54,55,56,57,58,59,60,61,62,63,64], in vitro using fluorescent proteins [45,65,66,67,68,69,70] and/or nanoparticles [71,72,73,74,75,76,77,78], and in amenable organisms such as chick embryos [79] and zebrafish [80,81,82], but translating FRET sensing to mammalian in vivo systems presents a number of challenges. First, the FRET sensor must be delivered to the site of interest. The mechanism of delivery varies significantly depending on the type of sensor being used. For example, genetically encoded FRET sensors using fluorescent proteins (FPs) as both the donor and acceptor have been extensively applied to cell culture systems [83], but there are fewer in vivo examples (such as [84,85]) because in vivo expression of the fluorescent proteins requires gene delivery (e.g., transgenic mice, injection with transfected cell lines, or gene therapy such as viral delivery or in vivo electroporation) and the visible light used for both excitation and emission of most FPs results in poor tissue-depth imaging.

Despite the challenges of in vivo FRET, multiple groups have pursued this approach because it provides one of the best opportunities to non-invasively monitor complex processes such as drug delivery [86,87], targeting [88,89], and release in mammals. In vivo FRET reports can be generally sorted into two categories based on the emission measurement modality. Historically, PL intensity-based measurements have dominated both in vitro and in vivo FRET [90,91]. Two color, ratiometric outputs are used for internal calibration, greatly reducing variances in the PL intensity based on fluorophore concentration [12,16]. More recently, photoluminescence lifetime measurements—the macroscopic equivalent of fluorescence lifetime imaging microscopy (FLIM)—have been adapted for in vivo imaging in mammalian systems. Using lifetime facilitates the elimination of background due to autofluorescence and can yield more information about the environment around the fluorescent sensor [92,93,94]. Both intensity and lifetime-based imaging have been used to monitor highly localized, microscale events taking place in the macrostructure of a whole organism [92,95,96]. 

**Figure 5:**
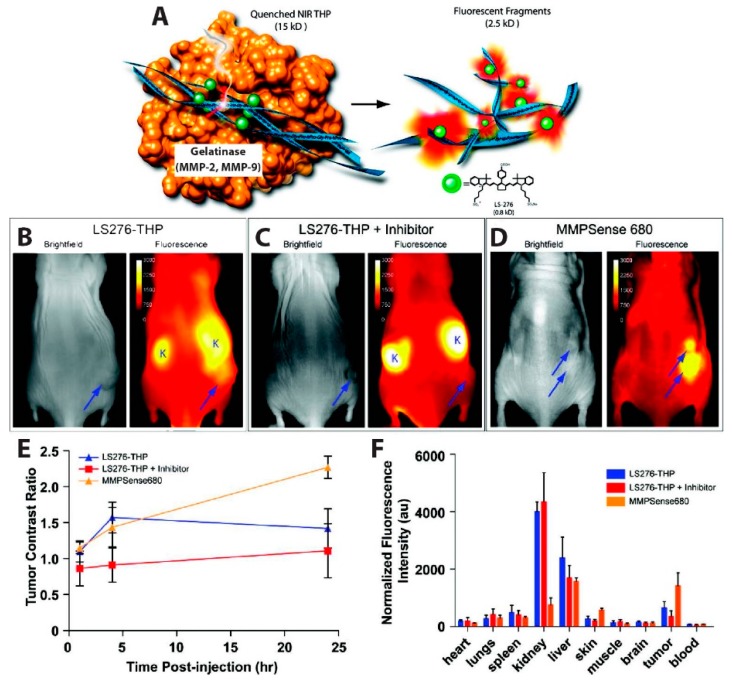
MMP overexpression in tumors detected with activatable NIR probe. (**A**) Schematic of the activatable probe. A type-V collagen sequence containing repeating Gly-Pro-4-hydroxy-l-proline Gly-Pro-4-hydroxy-l-proline (GPO) triplets conjugated to the NIR dye LS276 forms a triple helical peptide (THP) structure, quenching the dye emission through homoFRET. In the presence of MMP-2 or MMP-9, the THP is fragmented through enzymatic cleavage, resulting in enhanced brightness from the NIR probe. Images taken 24 h after i.p. injection of (**B**) LS276-THP; (**C**) LS276-THP with the MMP inhibitor Ilomastat; or (**D**) commercially available MMPSense 680. (**B**–**D**) The tumors are represented by an arrow and the kidney marked with the letter “K”. (**E**) Time-dependent evolution of NIR fluorescence shown by plotting the tumor contrast ratio, i.e., the ratio of the fluorescence intensity in the tumor and in a region of interest on the contralateral flank. (**F**) Plot of ex vivo fluorescence intensities by organ showing lower fluorescence intensity in the tumor when the inhibitor is present; other organs like the liver and kidney were not similarly impacted by the inhibitor. Fluorescence images were recorded at 830 ± 75 nm using excitation of 755 ± 35 nm for LS276 and 680 nm excitation and 720 nm emission for the MMPSense. Reprinted with permission from [110]. Copyright 2012 American Chemical Society.

The simplest iteration of an in vivo FRET device involves the self-quenching of a highly concentrated dye via homogenous FRET, or homoFRET. This kind of assay, which relies on the enhanced emission from the single-color dye when the local concentration of the dye is reduced, was used to visualize tumor-associated enzyme activity. Several matrix metalloproteinases (MMPs) are overexpressed in some human cancers, including breast [97,98,99], prostate [100,101,102], colorectal [103,104], and gastric cancer [105,106]. In this context, Akers et al., reported a highly specific activatable optical sensor for the in vivo detection of MMP-2 and MMP-9 activity in mice. LS276, a NIR fluorescent cyanine dye [107], was covalently labeled to the Lys residues flanking the hydrolysis site of a type-V collagen sequence GPPG↓VVGEKGEQ. This sequence was modified as described by Yu et al. to contain repeating Gly-Pro-4-hydroxy-L-proline (GPO) triplets at both the N- and C- termini, resulting in self-assembly into triple helical peptides [108]. The triple helical peptide (THP) structure forces multiple LS276 dyes into close proximity, inducing fluorescence quenching with efficiency of 73.5% ± 0.5%. Fluorescent signal was restored upon cleavage of the scissile bond (represented as ↓) by MMP-2 or MMP-9 (Figure 5A). In order to image in vivo MMP activity, the LS276-THP and a commercially available MMP imaging agent, MMPSenseTM 680, were interperitoneally (i.p.) injected into male NCR nude mice xenografted with HT1080 human fibrosarcoma cells known for their high MMP expression. MMPSenseTM 680 is a proprietary, large molecular weight (~450 kDa), protease-activated imaging agent sold by PerkinElmer. This large construct comprises many quenched dyes that fluoresce when they are released from the macromolecule following cleavage by MMP-2,-3,-9, and/or -13 [109]. Intraperitoneal (i.p.) injection was chosen because LS276-THP probes administered intravenously (i.v.) were rapidly cleared via the kidneys, due to the relatively small molecular weight (~15 kDa) of the sensor. Fluorescence images (Figure 5B–D) were recorded using an excitation wavelength of 755 ± 35 nm or 685 nm and detection at 830 ± 75 nm or 720 nm, for mice injected with LS276-THP or MMPSense 680, respectively. Figure 5E showed a rapid increase in fluorescence intensity 4 h post-injection, while a gradual increase and no rapid increase in fluorescence was observed in mice treated with Ilomastat which is an effective inhibitor of MMP activity. Their observations also indicated higher tumor activation (Figure 5D,E) when imaging with MMPSenseTM 680 than with LS276-THP. The authors attributed this difference to a lack of selectivity of MMPSenseTM 680, which can be cleaved by multiple MMP family proteases. Ex vivo fluorescence imaging (Figure 5F) indicates high signal intensities in the kidney and liver due to a mixed elimination route for the probe and, eventually, its hydrolyzed fragments. Fluorescence intensity was observed to be lower in the tumor containing the inhibitor as compared to the probes only. Immunohistochemistry of the removed tumor suggested cleavage of LS276-THP by MMP-2 [110].

**Figure 6:**
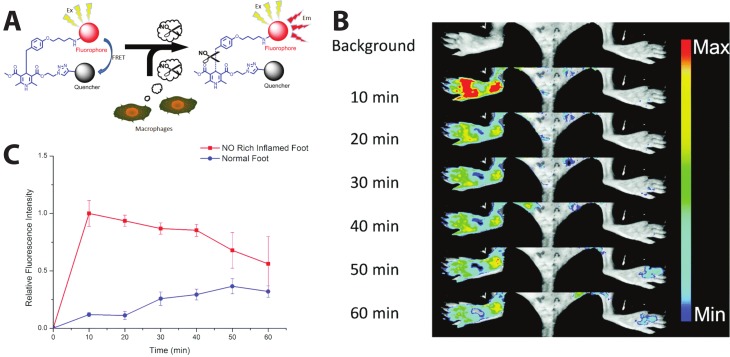
In vivo monitoring of inflammation-associated NO concentration. (**A**) Schematic of NO sensor. (**B**) Left foot treated with Freund’s adjuvant to induce inflammation; right foot is the inflammation-free control. (**C**) Comparison of the relative fluorescent intensity in the NO-rich inflamed foot and normal foot after intravenous injection of the probe. An 8-fold higher fluorescence intensity was observed in the inflamed area compared to the control area within 10 min post injection. Fluorescence signals were collected at 600 nm after 470 nm excitation. Reprinted from [114]. Distributed under a Creative Commons Attribution 3.0 Unported License (CC BY 3.0).

Extending the self-quenched homoFRET system, one can use a non-fluorescent quencher—i.e., a non-fluorescent chromophore whose absorbance exhibits high spectral overlap with the donor emission—as the FRET acceptor in another variant of a single-color, emission intensity-based probe. One such sensor was developed to visualize the presence of nitric oxide (NO), which is implicated in different physiological processes, such as immune responses [111] and nerve cell communication [112,113]. In order to image and detect NO as an inflammatory factor, Li et al. succeeded in sensing NO in vivo in mice using a cleavable FRET sensor. Their probe, named dihydropyridine-fluorophore-quencher (DHPFQ), is reportedly the first ‘turn on’ fluorescent probe to be used in mammals for the specific detection of NO. Here, the FRET donor, FITC, and a non-fluorescent acceptor, DABCYL, are linked with 1,4-dihydropyridine. When the sensor is intact, DABCYL quenches the FITC emission, but NO-induced cleavage of the C-C bond through homolysis between 1,4-dihydropyridine and a benzyl group at the C4-position irreversibly separates the donor and quencher. The probe exhibited high specificity for NO compared to other reactive oxygen or nitrogen species. Three days after a subcutaneous injection of Freund’s adjuvant to initiate inflammation on the left rear paws of mice, 0.5 mg kg^−1^ DHPFQ was injected intravenously and the mice imaged every ten minutes for one hour (Figure 6A). Using the semi-quantitative analysis of a region of interest (ROI) on the fluorescence image, their findings showed an 8-fold increase in fluorescence intensity of FITC within 10 min post-injection in the inflamed region of the left paw as compared to the non-inflamed paw, leading to nanomolar detection of NO (Figure 6B) [114].

**Figure 7:**
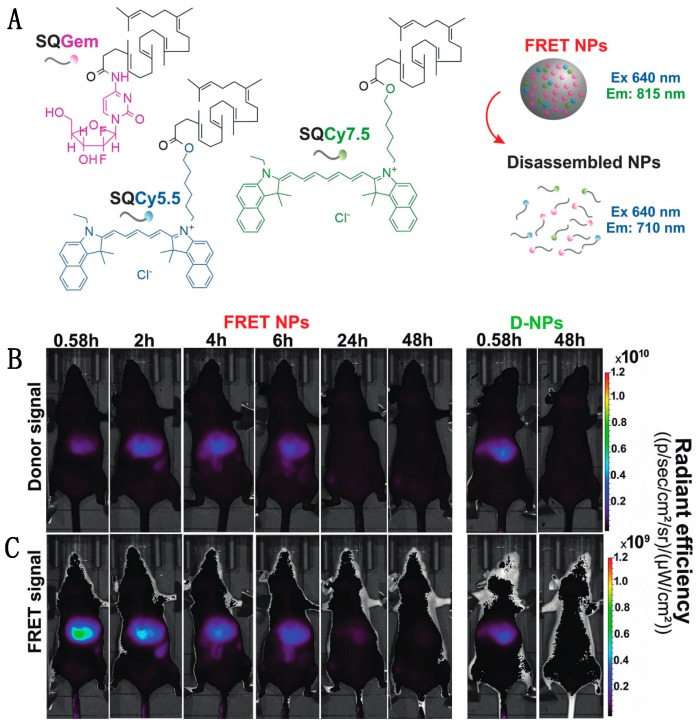
Using FRET to monitor the in vivo fate of NPs in the liver of mice following intravenous injection. (**A**) Self-assembly of SQGem, SQCy5.5 and SQCy7.5 to form a nanoparticle. Nanoparticle integrity verified by FRET by observing the transition from FRET NPs to disassembled NPs. (**B**) Donor channel signal collection at 697–770 nm. At 0.58 h, FRET NPs in this channel exhibited lower signal intensity than D-NPs, confirming FRET-based quenching. (**C**) FRET-induced acceptor emission collected between 810–875 nm. At 0.58 h, the high acceptor emission intensity indicates that the FRET NPs retained their integrity in the liver. Comparing (**B**,**C**) at 2 h post-injection, the increase in signal in the donor channel and a decrease in signal in the acceptor channel indicates disassembly of the NPs. The images demonstrate the timing of NP disassembly through the reduction in the FRET-induced acceptor emission intensity. Reproduced with permission from [87]. Copyright 2017 WILEY-VCH Verlag GmbH & Co. KGaA, Weinheim.

Though turn-on probes such as the NO sensor can be useful, fluorescence intensity is also impacted by other factors, such as concentration or pH fluctuations, which confounds the interpretation of single-color sensor data. In contrast, pairing an emissive donor to an emissive acceptor allows for a ratiometric output that internally calibrates for changes in concentration or other environmental factors. This kind of dual-color FRET-based imaging has been particularly useful in tracking the behavior of nanomedicine devices, such as nanoparticle-based drug delivery vehicles, even in mice. Cayre et al. used FRET to monitor the fate of nanocarriers based on the natural lipid squalene (SQ) in vivo in mice by labeling SQs with two dyes acting as a FRET pair as well as gemcitabine (Gem), a tumor chemotherapy prodrug [115]. As depicted in Figure 7A, the SQ-based nanoparticle forms through lipophilic self-assembly, co-localizing two dyes: Cy5.5 and Cy7.5 acting as FRET-donor and FRET-acceptor, respectively. After successful confirmation of the NP integrity through FRET, the dye-labeled SQ-Gem NPs were injected into the lateral tail vein of mice and imaged at different time intervals (Figure 7B,C). Following excitation at 640 nm, the donor emission and sensitized acceptor emission were collected at 695–770 nm and 810–875 nm, respectively. High FRET, as indicated by high intensity acceptor emission, indicated the presence of intact particles, while disassembled particles no longer held the two dyes in close proximity, reducing FRET. The imaging study showed maximum FRET-efficiency in the liver 35 min post-injection (0.58 h) with approximately 56% of NPs still intact; by 2 h post injection, a decrease in acceptor emission indicated < 10% NP integrity. The similar low-level emission observed in both the donor and acceptor channels of control group (D-NPs) contrasts with the low donor signal and high acceptor signal seen shortly after injection for the FRET NPs (Figure 7B,C, leftmost panels), demonstrating that the acceptor emission intensity was indeed a result of FRET-pair interactions. These findings suggest a rapid disassembly of these nanoparticles in the liver and elimination of squalene bioconjugates after 24 h with no hepatic buildup [87].

Similarly, the integrity of lipid nanocapsules (LNCs) was observed in vivo using FRET. Gravier et al. co-encapsulated hydrophobic dialkylcarbocyanine fluorophores, DiI and DiD, in a LNC lipid core and observed their stability in solution, after cell internalization, and in vivo [116]. The in vivo fate of LNCs was analyzed by intravenously injecting them in mice bearing subcutaneous TS/A-pc tumors; this mouse mammary carcinoma line is known for its high enhanced permeability and retention (EPR) effect. At three time points post-injection (1, 5, and 24 h), ratiometric imaging of two regions of interest around the tumor and liver indicated that LNC found in the liver were more likely to be dissociated than LNC in the tumor region. Specifically, whole animal imaging produced autofluorescence-subtracted acceptor emission intensity to donor emission intensity ratios of 11.1 ± 0.3 and 17.2 ± 1.5 in the liver and tumor, respectively, 24 h post-injection. The authors indicate that the aggressive environment in the liver degrades the LNCs more rapidly than the tumor environment. Ex vivo confocal microscopy images confirm decreasing acceptor emission intensity in the tumor with the authors estimating that the fraction of intact LNCs decreases from 39% 1 h post-injection to 11% after 5 h and 6% after 24 h. Their findings nicely show that FRET measurements can reveal information about the integrity of nanocarriers in a tumor environment and how their content is released over time, while also highlighting the need to concomitantly assess the fate of nanomaterials in the liver [116].

In another example, the dissociation and tumor accumulation dynamics of self-assembled lipidic nanoparticles (SALNPs) was studied by Zhao et al. in vivo in a mouse model using QDs and fluorescent dyes. Their SALNPs were composed of QDs coated by a PEGylated self-assembled lipid monolayer and dye-lipids. FRET pairs combined QDs with emission peaks at 610 nm or 710 nm (QD610 or QD710, FRET donors) and Cy5.5 or Cy7 emitting around 710 nm or 800 nm (FRET acceptors) to produce SALNPs denoted QD610-Cy5.5-PEG and QD710-Cy7-PEG. After intravenous (i.v) injection of QD710-Cy7-PEG into a nude mouse bearing a HCT116 colon carcinoma on its flank, FRET intensities recorded at 800 ± 10 nm (after excitation at 605 ± 18 nm) drastically decreased 48 h post-injection, confirming the dissociation of Cy7-lipids from QD710 after tumor accumulation. Using an intravital confocal laser scanning microscopy technique on tumors grown in dorsal window chambers in mice, the authors determined the dissociation kinetics of SALNPs in the tumor blood vessels and tumor interstitium. Two hours post-i.v injection of QD610-Cy5.5-PEG, the authors observed a decrease in the FRET/QD ratio in the vascular and extravascular space caused by the dissociation of Cy5.5-lipids from the SALNPs. The lipid dissociation constant from the vascular space was determined to be 2.7 × 10^−4^ s^−1^ with a dissociation half-life of approximately 42 min [117].

In vivo FRET imaging using visible dyes suffers from reduced sensitivity due to the poor penetration of light through highly scattering and absorbing biological tissues. Switching to NIR-emitting dyes can help significantly, as both excitation and emission wavelengths are red-shifted and generally exhibit improved tissue penetration. Alternatively, issues of light penetration can be addressed using upconverting nanoparticles (UCNPs). UCNPs are core or core/shell nanoparticles composed of optically active sensitizer and activator lanthanide ions doped into host lattices of chlorides, bromides, or iodides [118,119]. The sensitizer (e.g., Yb^3+^ or Nd^3+^) is responsible for absorbing NIR excitation light while the activator (e.g., Er^3+^, Tm^3+^, or Ho^3+^) acts as the emitting ion [118]. Due to the increased transparency of biomolecular structures in the optical tissue window, NIR excitation of the UCNPs greatly reduces autofluorescence and provides deeper penetration depth. Detection of emitted light from biological tissues can therefore be recorded with high signal-to-background ratios [120]. A review on the nanotoxicity of UCNPs by Gnach et al. indicated that reports to-date indicate low toxicity, but that more reports on their long-term impact is needed [121].

**Figure 8:**
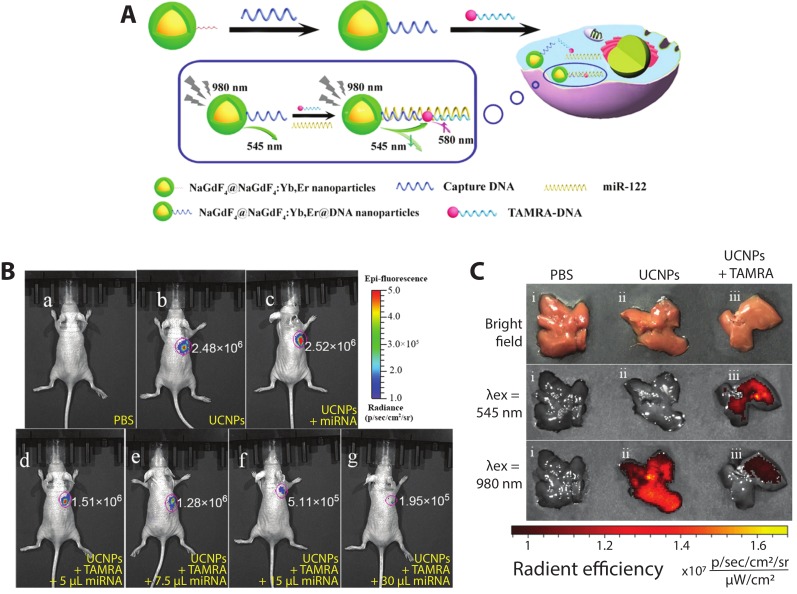
Fluorescence detection of miRNA-122 using a nucleic acid hybridization-based FRET construct with UCNPs. (**A**) Schematic of the hybridization assay. DNA-labeled UCNPs and DNA-labeled TAMRA are brought into close proximity upon binding to distinct complimentary regions of miRNA-122. Following excitation at 980 nm, the upconversion emission of the UCNP at 545 nm is transferred to the nearby TAMRA dye via FRET, reducing the emission at 545 nm and inducing sensitized emission at 580 nm. (**B**) Nude mice with human liver cancer HepG2 cells with subcutaneous injection of (**a**) 100 µL PBS; (**b**) 50 µL of 20 mg/mL captureDNA-UCNPs + 50 µL of 5 µM DNA-TAMRA; (**c**) 50 µL of captureDNA-UCNPs + 30 µL of 5µM miRNA-122; (**d**) 50 µL of captureDNA-UCNPs + 50 µL of DNA-TAMRA + 5 µL of 5 µM miR-122; same concentrations of probe + (**e**) 7.5 µL miRNA-122 (**f**) 15 µL miRNA-122 (**g**) 30µL miRNA-122. (**C**) Healthy livers of nude mice after tail vein injection of 200 µL of PBS; captureDNA-UCNPs + 100 µL PBS; and 100 µL of 20 mg/mL captureDNA-UCNPs + 100 µL of captureDNA-TAMRA. Adapted with permission from [88]. Copyright 2018, American Chemical Society.

Recently, core–shell upconverting nanoparticles (UCNPs) were used for the in vitro and in vivo detection of miRNA-122, which is thought to be a crucial regulator of cholesterol metabolism in the liver [122] as well as a potential nucleic acid biomarker for liver cancers [123]. Ren et al. constructed a DNA hybridization-based FRET system (Figure 8A) with a core–shell UCNP (core—NaGdF_4_; shell—NaGdF_4_:Yb,Er) and TAMRA dye, each functionalized with a DNA sequence complementary to different regions of miRNA-122. In the presence of miRNA-122, DNA hybridization brings the UCNP and TAMRA dyes into close proximity such that, following excitation at 980 nm, the 545 nm UCNP emission is quenched and sensitized TAMRA emission emerges at 580 nm. In vitro testing of the sensor yielded a limit of detection of 10^−13^ M miRNA-122 and FRET efficiency of 49%. To detect exogenous miRNA-122 in vivo, mice with subcutaneously xenografted human liver cancer HepG2 cells, which exhibit downregulation of miRNA-122, were injected with various probes and controls, including exogenous miRNA-122. As visualized in Figure 8B(b,c), no visible difference in intensities can be observed in mice injected with UCNPs + TAMRA and UCNPs + miR-122, showing the necessity of all three components for the DNA hybridization-based FRET to occur. In mice injected with UCNPs + TAMRA + different concentrations of miRNA-122 (Figure 8B(d–g)), the fluorescent signal decreased by up to an order of magnitude with increasing amounts of miRNA-122. Endogenous miRNA-122 were then detected in healthy mice following tail vein injection of different probes and controls. At 4 h post-injection, the mouse livers were imaged ex vivo (Figure 8C). In the PBS control, no fluorescence is detected; when the UCNPs were dosed via tail vein injection, bright emission is seen following 980 nm excitation; when the DNA-labeled UCNPs and DNA-labeled TAMRA are co-injected, emission from the UCNPs is substantially curtailed. Emission following excitation at 545 nm confirms that the TAMRA is present in the liver, supporting the hypothesis that the miRNA-122 binding-mediated FRET quenches the UCNP upconversion emission [88]. The imaging of exogenous and endogenous miRNA by this approach shows its effectiveness for in vivo FRET biosensing.

Excitation of UCNPs at 980 nm, as in the studies above, raises concerns about heating effects in biological samples as this wavelength is highly absorbed by water [124,125]. To mitigate this, Zou et al. built sensors using UCNPs with Nd^3+^ as the sensitizer rather than Yb^3+^, enabling excitation at 808 nm [126]. Their sensor for hypochlorite (ClO^−^) was motivated by the relevance of hypochlorite overproduction to many human diseases such as lung injury [127], rheumatoid arthritis [128], and cancer [129]. The Nd^3+^-doped UCNPs produce upconversion emission at 540 nm and 655 nm; the cyanine dye hCy3 was used as a FRET-acceptor to the 540 nm emission, as structural changes to the dye in the presence of ClO^−^ cause its absorption cross-section to decrease in response to ClO^−^ concentration. Coupling the UCNP with hCy3 produces a turn-on probe with increasing upconversion emission at 540 nm in response to higher ClO^−^ concentration and lower hCy3 absorption. Due to this effect, a ratiometric measurement of upconverting emission at 540 and 654 nm was selected to quantify ClO^−^. Increasing concentration from 0 to 80 µM of NaClO with hCy3-cs-UCNP:Nd resulted in a linear response in the ratio of upconversion emission and a limit of detection of 27 ppb, which is equivalent to 500 nM. The sensor was used for in vivo imaging of ClO^−^ production in a 4-week-old mouse with arthritis induced in the left ankles with local injections of λ-carrageenan. After 4 h, hCy3-csUCNP:Nd were injected into both ankles and upconverting luminescence was acquired at 530 ± 25 nm after 808 nm excitation. The control ankle exhibited 2-fold lower emission compared to the inflamed region, indicating the ability of the sensor to detect arthritis-dependent ClO^−^ production in vivo [126].

**Figure 9:**
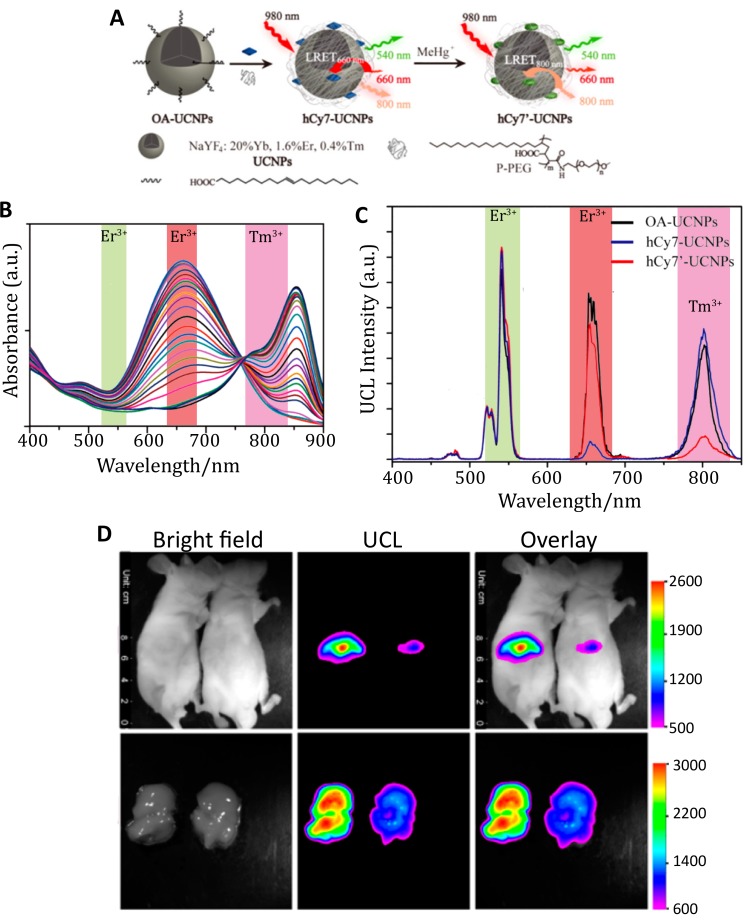
Detection of MeHg^+^ accumulated in mouse livers in vivo using hCy7-UCNPs. (**A**) Schematic of the hCy7-UCNP probe. UCNPs decorated with hCy7 exhibit quenching of their emission at 660 nm; exposure to MeHg^+^ converts hCy7 to hCy7’, which quenches the UCNP emission at 800 nm instead. (**B**) Titration of MeHg^+^ to hCy7 reduces the absorbance peak at 670 nm while enhancing absorbance centered at 845 nm. In this titration, the MeHg^+^ concentration was titrated from 0 to 56 µM. The colored bands indicate overlap with the emission peaks from the UCNPs. (**C**) Upconversion emission of the UCNPs (black) is present in three major bands around 540 nm (from Er^3+^), 660 nm (from Er^3+^), and 800 nm (from Tm^3+^). In the absence MeHg^+^, the peak at 660 nm is quenched by hCy7 (blue). In the presence of MeHg^+^, the peak at 800 nm is quenched by hCy7’ (red). (**D**) In vivo imaging of hCy7-UCNPs. Top: Mice were IV injected with 40 µg of the hCy7-UCNP probes followed by IV injection of 200 µL 0.1 mM MeHg^+^ solution (right mouse) or saline solution (left mouse). Luminescent images recorded at 800 ± 12 nm after 980 nm excitation indicate 50% quenching of the upconversion luminescence (UCL) at 800 nm observed for the mouse treated with MeHg^+^. Bottom: Ex vivo images of the liver confirming accumulation and quenching of the probe. Adapted with permission from [131]. Copyright 2013, American Chemical Society.

The change in the optical properties of an organic dye in response to its environment was used again for UCNP-based FRET detection of methylmercury (MeHg^+^), a toxic ion whose accumulation into internal organs can result in organ damage, such as liver injury [130,131]. The heptamethine cyanine dye hCy7 exhibits a substantial dose-dependent red shift in its absorption peak from 670 nm to 845 nm in response to MeHg^+^ (Figure 9B) [131]. These two absorption peaks overlap with red Er^3+^ emission at 660 nm and NIR Tm^3+^ emission at 800 nm. By conjugating hCy7 to UCNPs comprising NaYF_4_:Tb:Er:Tm, the hCy7 absorbance could act as a FRET acceptor to Er^3+^ or Tm^3+^ emission in the absence or presence of MeHg^+^, respectively. The ratio of the red and NIR emission intensities yielded an internally calibrated sensor output with a limit of detection of 0.18 ppb in solution. In vivo monitoring of MeHg^+^ was demonstrated by i.v. injecting hCy7-UCNPs in mice followed by either MeHg^+^ or a control solution. Upconversion images acquired at 800 ± 12 nm after 980 nm excitation demonstrate that hCy7-UCNPs accumulated principally in the liver (Figure 9D). About 50% quenching of the upconversion emission at 800 nm was observed when compared to the control group, as confirmed with ex vivo imaging [131]. Adoption of in vivo ratiometric imaging of this sensor, as was demonstrated spectrally, would nicely enhance the utility of this kind of indicator independent of the direct comparison to the control.

Each of the FRET examples above relies on changes in the luminescence intensity as the signal output. Photoluminescence lifetime is a complimentary and powerful tool for imaging because the lifetime is dependent on the environment and excitation mechanism of the fluorophore, but is independent of dye concentration, assuming the fluorophore is dilute enough to avoid homoFRET and other near-field effects [132,133]. Each fluorophore exhibits its own characteristic PL lifetime, which can range from ps for organic dyes to µs for lanthanide-based emitters [134]. In a FRET construct, the donor lifetime is shortened by efficient energy transfer, as energy is siphoned off by the acceptor. Concomitantly, the acceptor fluorophore exhibits what appears to be a lengthened PL lifetime; in reality, the characteristic lifetime of the acceptor is not changed, but the injection of excitation energy comes later as the process of donor excitation and energy transfer takes a measurable amount of time [11]. In this context, one can observe FRET through the measurement of the donor and/or acceptor lifetime. FRET has been combined with fluorescence lifetime imaging microscopy (FLIM) in vitro for FRET-FLIM-based observations of nanoscale interactions [133]. On the macroscale, this was demonstrated by McGinty et al. using a FP-FP FRET pair to image the hindleg muscles in live mice expressing GCLink, a FRET probe composed of an eGFP FRET donor fused to an mCherry FRET acceptor. GCLink-containing plasmids (1.0 µg/µL) as well as plasmids co-expressing unlinked eGFP (1.5 µg/µL) and mCherry (1.5 µg/µL) were transfected by electroporation into the right hind leg of female mice in order to target the tibialis anterior (TA) muscle. Using time-gated imaging, eGFP was excited at 480 ± 20 nm with ~10 ps pulses and the emitted fluorescence recorded using a CCD camera. The authors successfully imaged the hind legs of mice expressing GCLink, as well as free eGFP and mCherry, by reconstructing the fluorescence lifetime distributions of eGFP. The reconstructed lifetimes of eGFP in the TA muscle revealed a decrease of fluorescence lifetime in mice expressing GCLink compared to control mice with mean eGFP lifetimes of 1.3 ± 0.2 ns and 2.2 ± 0.2 ns, respectively. They also showed that tissue autofluorescence did not cause any significant changes in eGFP lifetime reconstruction as the eGFP signal was ~200-fold higher than the autofluorescence signal when measured at 530 ± 20 nm [135]. The FRET between the eGFP and mCherry was not used to study a particular biological or pharmacological event, but rather was used to demonstrate the power of using advanced instrumentation and data analysis approaches for generating a clearer picture of biomolecular interactions in vivo.

In a rather elegant example of how macroscale FRET-FLIM can be used to differentiate complex biological processes in vivo, lifetime measurements of two NIR-emitting dyes were used to discern specific and non-specific payload uptake in a tumor [89]. While the enhanced permeability and retention (EPR) effect can be used to successfully deliver nanosized payloads to leaky tumors, discerning between non-specific EPR-based delivery and specific, targeted delivery is a significant challenge. To non-invasively differentiate between receptor-bound ligands and non-specifically accumulated ligands in tumors in vivo, Abe et al. developed a novel FRET imaging method based on monitoring fluorescence lifetime [89]. In this system, overexpressed transferrin receptors (TfnR) were tagged with the native ligand transferrin (Tfn). Typically, two Tfn bind homodimerized TfnR, resulting in binding-specific colocalization of targeted moieties. Using batches of Tfn conjugated to the FRET donor Alexa Fluor 700 (AF700) and FRET acceptor AF750, binding of two Tfns to a TfnR homodimer brings the two dye-labeled ligands within 2–10 nm of each other, inducing FRET. Bound ligands exhibited a shortened donor PL lifetime, while the lifetime was not shortened by FRET for unbound ligands. Measuring the PL lifetime rather than emission intensity has a distinct advantage as the fluorescence intensity can be decreased by changes in local concentration and environmental effects (e.g., pH, temperature) as well as FRET [132]; the use of PL lifetime is more reliable for quantification. Nude mice inoculated with palpable, orthotopic T47D breast tumors were dosed with tail vein injections of AF700- and AF750-conjugated Tfn at acceptor:donor ratios of 0:1 and 2:1. At 1-h post injection, the proportion of Tfn-Tfn-TfnR complexes, denoted as FRET donor fraction (%FD), at the plasma membrane and in the intracellular membranes could be discerned in live animal lifetime imaging. Subsequently, they could also discriminate the population not involved in FRET by determining the non-FRET donor fraction (%NFD), which constitutes extracellular and unbound AF700-Tfn. From analysis of the fluorescence signals, the authors showed that with an acceptor:donor ratio of 2:1, a decrease in the photoluminescence lifetime of AF700 was observed compared to the 0:1 ratio (i.e., AF700-Tfn only) in vivo. This resulted in an increase in %FD, suggesting that approximately 30% of the total Tfn injected was bound and internalized by the tumor cells in mice [89].

Another example of using advanced imaging approaches for FRET uses fluorescence anisotropy as a means to discern the close proximity of multiple fluorophores of the same type. In this case, homoFRET is observed not in the form of self-quenching, but rather by measuring the polarization of the emitted light following excitation with polarized light. This approach has been demonstrated in vivo in the observation of actin polymerization in situ in mice brains. When actin-GFP monomers were incorporated into endogenous actin polymers, homoFRET (also termed energy migration FRET or emFRET in this report) between neighboring actin-GFPs substantially lowered the polarization of the emitted light compared to that of direct emission from GFP molecules not participating in emFRET. By deriving a relationship between the fluorescence anisotropy of the GFP and the fraction of actin polymerized, the authors were able to image the actin polymerization state with high spatiotemporal resolution in vivo. While the resolution was enhanced through the use of two-photon imaging, steady-state one-photon fluorescence anisotropy imaging could be used as well [136].

## 4. In Vivo BRET

Through BRET (and CRET, as described below), energy transfer is used for sensing and/or to facilitate imaging itself through near-field excitation. The use of molecular reactions as a source of incident excitation eliminates the need for photoexcitation, mitigating several limitations of photoluminescence: (1) blue and green light generated through bioluminescence can be used locally without concerns about tissue attenuation; (2) potentially damaging effects of high intensity excitation light are avoided (e.g., UV photodamage or heating from NIR light); and (3) autofluorescence from photoexcitation of tissue is avoided; the extremely low background signal significantly increases signal-to-noise ratios [137,138]. It should be noted, however, that while photoexcitation is no longer needed, researchers instead need to ensure that the bioluminescent substrate is efficiently delivered to the bioluminescent enzyme, as the availability of this molecule becomes the limiting component of the luminescent system.

In multiple examples, the bioluminescent reaction between luciferases and their substrates is used as a local source of excitation for secondary fluorophores. Emission from the secondary fluorophore is used as a beacon: for example, indicating tumor proliferation. Genetically engineered BRET constructs are particularly effective for monitoring tumor growth and metastasis; in these cases, the bioluminescent markers are propagated as cells divide and tumors metastasize, eliminating the need for subsequent probe delivery [139]. For the sensitive monitoring of tumorigenesis through in vivo imaging in mice using fluorescent protein-NanoLuc fusions as BRET pairs, Schaub et al., designed BRET-systems composed of the highly catalytic, blue-emitting luciferase NanoLuc and enhanced green fluorescent protein (eGFP), or long Stokes shift mOrange (LSSmOrange) [140]. The genetically encodable BRET reporters eGFP-NanoLuc (GpNLuc) and LSSmOrange-NanoLuc (OgNLuc) were dubbed LumiFluors. The GpNLuc LumiFluor was successfully expressed in A549 non-small cell lung cancer (NSCLC) and *Eµ-Myc* lymphoma cells. Xenografts of A549-GpNLuc NSCLC and A549-LKB1-GpNLuc NSCLC cells, where LKB1 protein is a tumor suppressor, were subcutaneously injected into the rear and front flanks of *NOD*/*SCID* mice, which were then imaged with bioluminescence imaging (BLI) after i.p. injection of furimazine. The data show that GpNLuc was sensitive enough to highlight 500 GpNLuc-expressing cells on day 1 after subcutaneous injection. In addition, tumor growth could be monitored over time, revealing that LKB1 suppressed NSCLC tumor growth after 28 days. Subsequent experiments demonstrated the ability to use the bioluminescence signal to visualize orthotopic tumors in deep lung tissues, as well as to observe hematological malignancies arising from lymphoma cells expressing FpNLuc [140].

The use of the genetically encoded LumiFluors was a clever approach to observe tumorigenesis and track malignancy formation: through genetic labeling, all generations of the primary and secondary tumors were luminescent, while the BRET construct combined bioluminescent excitation and red-shifted emission to improve signal and light penetration. Comparison of multiple LumiFluor constructs demonstrated that greatly extended exposure times were necessary to collect signal over background from the NanoLuc luciferase alone or NanoLuc chimeras with a non-fluorescent eGPF variant (25 and 40 s, respectively), while BRET from NanoLuc to eGFP or LSSmOrange produced wavelengths that penetrated the tissue more effectively, reducing exposure times to 6 and 3 s, respectively. Due to the red-shifted emission allowing deeper light penetration in tissues of OgNLuc, a 2–4-fold increase in BRET signal was observed relative to GpNLuc. Quantitative analysis of the acquired images showed an impressive signal-to-noise ratio 3–4 orders of magnitude higher than the signal produced in control mice injected with the substrate furimazine [140].

**Figure 10:**
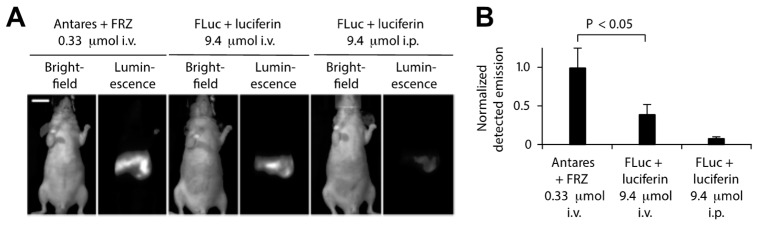
In vivo comparison of Antares and FLuc BRET emission intensities using the substrates furimazine and luciferin, respectively. (**A**) Antares results in a higher BRET emission when intravenously injected with 0.33 µmol furimazine (FRZ) as compared to FLuc when injected i.v. or i.p. with 9.4 µmol of luciferin. (**B**) Normalized emission from Antares and FLuc from multiple mice; n = 5 for Antares; n = 6 for FLuc i.v. and n = 16 for FLuc i.p. Adapted with permission from [141]. Copyright 2016, Springer Nature.

Additional efforts have been made to enhance the in vivo BRET imaging signal by modifying the BRET donor, BRET acceptor, and substrate properties as well as BRET efficiency and brightness. Coupling luciferase variants with redder emitting fluorescent proteins has been helpful, as long as the absorption spectrum still overlaps with the bioluminescence emission peak for efficient BRET. Chu et al., for example, developed an orange-red fluorescent protein CyOFP1 with peak emission at 589 nm and photoluminescent quantum yield of 76% to act as a BRET acceptor for NLuc [141]. BRET efficiency and CyPFP1 emission were optimized by modifying the linker between the two proteins and fusing CyPFP1 to both the C- and N-termini of NanoLuc, improving BRET by ca. 2-fold in the new chimera called Antares. Antares was observed to generate brighter bioluminescence signals with lower concentrations of substrate compared to the most commonly used BLI reporter firefly luciferase (FLuc) (Figure 10) [141].

Pushing BRET emitters into the NIR, Rumyantsev et al. designed fusions of RLuc8 luciferase with iRFP670 and iRFP720, which are fluorescent proteins with emission maxima at 670 nm and 720 nm, respectively, for deep tissue imaging and monitoring of tumor growth and metastasis in mice [142]. By constructing a calibration curve of the bioluminescence or fluorescence signal as a function of the number of cells, results showed that 10^3^ to 10^4^ cells could be detected subcutaneously by BLI, which was 10 times fewer than needed for detection with fluorescence imaging (FLI). A similar calibration was performed for deep tissue imaging, yielding a BLI limit of detection comparable to sensitive optical techniques such as fluorescence-lifetime imaging microscopy (FLIM) and reversibly switchable photoacoustic computer tomography (RS-PACT). After proving that the BRET construct was suitable for deep tissue imaging, tumor growth of orthotopically implanted xenograft breast tumors expressing iRFP670-RLuc8 or iRFP720-RLuc8 was monitored for 29 days. The deep tissue calibration curve was furthermore used to quantify tumor metastasis to the lungs using BLI. Finally, multiplexed imaging using both constructs in a single mouse indicated that this approach could be used to track multiple cancer lines in an organism simultaneously [142].

In an interesting BRET approach that mirrors the native aequorin to GFP energy transfer observed in jellyfish, a fluorescent probe was developed for in vivo imaging of calcium ions. Ca^2+^ is involved in many cell signaling pathways; its optical detection in vivo would facilitate the understanding of biological processes, including muscle contraction, cardiac function, neuronal stimulation [143]. Curie et al. fused the naturally Ca^2+^-sensitive blue-emitting aequorin to fluorescent proteins for the detection of Ca^2+^ in mice. Fusion proteins comprising GFP, the YFP mutant Venus, or monomeric red fluorescent protein 1 and aequorin (dubbed GA, VA, and RA, respectively) exhibited BRET in the presence CLZN and free Ca^2+^, red-shifting the blue aequorin emission via energy transfer to the fluorescent proteins. Light penetration of the GA, VA, and RA probes through different tissues —subcutaneous, subthoracic, or subcranial—was tested by placing small transparent plastic tubes containing the luminescent proteins, free Ca^2+^, and CLZN in different tissue sites in euthanized mice. Compared to control tubes adjacent to the mouse, 80% of light emission from VA penetrated through the skin with 71% and 40% from RA and GA, respectively. In the deeper site within the thoracic cage, <6% of BRET emission was transmitted for all three probes. Light transmitted through the mouse skull was further attenuated for GA (0.005%) and VA (0.02%) compared to the thoracic cage, but for RA exhibited similar light emission from both of the deeper tissue sites (4.9% in subthoracic tissue vs. 4.6% in subcranial tissue) [144]. This study shows that the aequorin-FP reporters could be used to non-invasively image Ca^2+^ activity in the heart and brain and to study Ca^2+^ dynamics. In deeper tissues, the red emission was able to penetrate more effectively, but at more superficial depths, VA was more effective because of the large spectral overlap between the absorption spectrum of Venus and the emission spectrum of aequorin, yielding more efficient BRET compared to GA or RA. In another paper from the same group, transgenic mice expressing a subcellularly localized GA construct facilitated whole animal imaging of Ca^2+^ concentration in the mitochondrial matrix ([Ca^2+^]_m_). The utility of whole-body [Ca^2+^]_m_ imaging was demonstrated in a study of sleep/wake cycle states (including whole body startle, coordinated movement, and atonia) in freely moving newborn mice [145].

**Figure 11:**
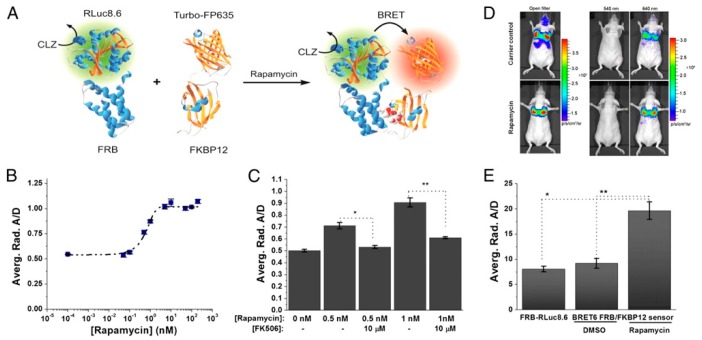
BRET sensor for protein–protein interactions in the lungs of mice. (**A**) Schematic of the BRET system. The proteins FRB and FKBP12 form a heterodimer upon addition of the small molecule rapamycin. As the FRB and FKB12 used are chimeras with the luciferase RLuc8.6 and the fluorescent protein Turbo-FP635, respectively, the dimerization brings the BRET donor and acceptor into close proximity, facilitating energy transfer. (**B**) Dose–response curve of 1 × 10^5^ HT1080 cells expressing the BRET-CID system 6 h after addition of rapamycin with an estimated EC_50_ of 0.7 ± 0.2 nM. (**C**) Influence of the inhibitor FK506 on the BRET ratios demonstrating the specificity of the BRET6 sensor. (**D**) Bioluminescence images of control (top) and rapamycin-dosed mice (bottom) after CLZ injection using open, donor, and acceptor filters. (**E**) Comparison of the BRET-ratios of control groups, which include the BRET donor only (FRB-RLuc8.6) and the donor–acceptor pair in the absence of the CID-inducer (BRET6 FRB/FKBP12 sensor, DMSO), with the BRET donor–acceptor pair in the presence of the CID-inducer (BRET6 FRB/FKBP12 sensor, rapamycin) [146]. Copyright 2011 Dragulescu-Andrasi et al.

Dragulescu-Andrasi et al. extended the premise of using Luc-FP BRET for in vivo sensing considerably by using Luc-FP BRET to image protein–protein interactions in mice [146]. The ligand-mediated association of the two protein halves of the classic chemically inducible dimerization (CID) pair FK506 binding protein 12 (FKBP12) and FKBP12-rapamycin binding domain (FRB) has been used as a prototypical protein–protein interaction for the development and testing of a variety of biosensors [147,148] and was extended to an in vivo BRET system as well. First, the group tested several BRET donor–acceptor pairs and substrates and optimized a ratiometric imaging technique, termed double ratio, that yielded an internally calibrated BRET signal that partly corrects for the wavelength-dependent emission photon attenuation in tissue and is independent of the number of cells expressing the BRET reporter. The ratiometric BRET output also integrates an internal control, mitigating changes in luminescence intensity based on variations in reporter concentration. Next, the CID system was developed using the optimized BRET pair. Specifically, fusion proteins of FRB and FKBP12 with RLuc8.6 and TurboFP635—the BRET donor and acceptor, respectively—were expressed in a fibrosarcoma cell line (HT1080) and subsequently i.v. injected into nude mice. The tumor cells migrated to and implanted in the mice lungs, where protein–protein interactions could be observed (Figure 11). Upon addition of rapamycin, the FRB-FKBP12 molecular interaction brought the RLuc8.6 and TurboFP635 into close proximity, inducing BRET. BLI of the mice showed BRET signal in the lungs with a 2.4-fold higher BRET ratio for the rapamycin-dosed mice compared to controls. Specificity of the sensor was also confirmed using FK506, an inhibitor for the rapamycin-FRB-FKBP12 association. These findings showed the benefits of using ratiometric imaging in vivo and the ability to monitor protein–protein interactions through resonance energy transfer techniques in deep tissues, such as directly in the lungs of disease models in mice [146].

**Figure 12:**
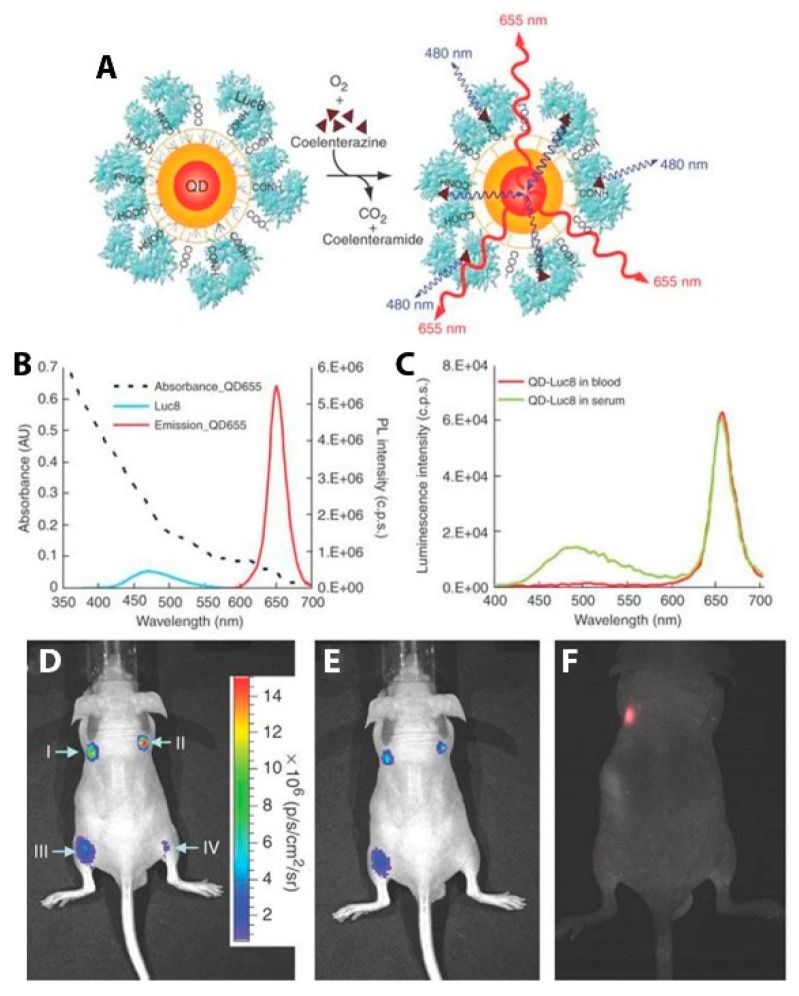
In vivo BRET-based imaging of labeled C6 glioma cells in mice. (**A**) Schematic of a QD covalently coupled to the BRET donor, RLuc8. The bioluminescence energy of RLuc8-catalyzed oxidation of coelenterazine is transferred to the quantum dots, resulting in QD emission. (**B**) Absorption and emission spectra of QD655 (λ_ex_ = 480 nm) and spectrum of the bioluminescent light emitted in the oxidation of coelenterazine catalyzed by RLuc8. (**C**) Bioluminescence emission spectrum of QD655-Luc8 in mouse serum and in mouse whole blood. (**D**–**F**) Bioluminescence and fluorescence imaging of QD655-Luc8 and Luc8 injected subcutaneously (I and II) and intramuscularly (III and IV) at indicated sites in a mouse (I and III, QD655-Luc8, 5 pmol; II and IV, Luc8, 30 pmol). (**D**) Bioluminescence image taken without filters. (**E**) Bioluminescence image taken with 575-650-nm filter. (**F**) Fluorescence imaging of the same mouse using 503–555 nm excitation filter. Adapted by permission from [26]. Copyright 2006, Springer Nature.

In addition to FPs, semiconductor QDs have been particularly successful in the role of the acceptor fluorophore in BRET imaging constructs for several reasons: (1) the overlap integral between luciferase and the QDs is exceptionally high because of the broad absorbance spectrum and high absorption cross-section of the QD; (2) multiple luciferases can be attached to a single QD, increasing the chances that any given QD is emitting; (3) the large effective Stokes shift of the QD allows for efficient energy transfer from blue-emitting luciferases and optimal emission light penetration with red or NIR QD emitters [26]. An early example of QD-based BRET imaging demonstrated the benefits to light penetration depth seen using the local BRET-based excitation of QD contrast agents. Self-illuminating QDs developed by So et al. were used to pre-label C6 glioma cells that accumulated in the lungs of nude mice after tail vein injection. Using EDC-based bioconjugation, polymer-coated CdSe/ZnS QDs emitting at 655 nm (QD655) were decorated with RLuc8 (Figure 12A). The particles were internalized by cultured C6 glioma cells, which were then injected into nude mice, where they localized to the lungs. After i.v. injection of the bioluminescent substrate CLZN, the QD-labeled cells could be seen in two distinct lung lobes (Figure 12B). Controls ensure that the luminescence detected was from the QDs and not just the luciferase. Direct excitation of the QDs through the animal tissue with 503–555 nm light did not yield signal from the lungs due to absorption and scattering of the excitation light (Figure 12C). These self-illuminating QD conjugates demonstrate the advantage of circumventing external excitation in deep tissue imaging as a means to minimize biological sample autofluorescence and maximize detection sensitivity in vivo [26].

Subsequent iterations of BRET-based imaging with QDs facilitated in vivo lymphatic imaging [149,150] and targeted tumor imaging [150], reliably reducing autofluorescence compared to epifluorescence imaging. In one comparison of tumor-to-background luminescence ratios for both fluorescence imaging and bioluminescence imaging, images of subcutaneous U87MG human glioblastoma tumors were 4× brighter than background in fluorescence modes, but 90× brighter in bioluminescence mode, demonstrating the superior sensitivity of BRET-based excitation [150]. Xing et al. demonstrated that polymeric encapsulation of QD-Luc8 BRET conjugates improved in vivo stability for prolonged bioluminescent imaging in mice [151]. The BRET-based self-illuminating QDs were turned into protease sensors though the incorporation of a peptide cleavage sequence between the QD and the luciferase. The BRET sensor improved sensitivity to enzyme activity by several orders of magnitude compared to contemporaneous QD-FRET and magnetic bead-based sensors in vitro, was effective in complex media, and exhibited multiplexed detection of proteases, but does not appear to have been demonstrated in vivo [152,153].

In addition to imaging and sensing, BRET has been adapted for local photoinduction in vivo. For example, BRET has been cleverly harnessed as a way to use bioluminescence as a local light source for optogenetics by several labs [154,155]. These novel constructs involve fusing a light-emitting luciferase to an optogenetic ion channel, thus forming a luminescent opsin, dubbed ‘lumiopsin’ (LMO). These LMOs, paired with standard optogenetic tools, allow for multi-modal control of neuron simulation: i.e., rapid modulation of a subset of neurons by optogenetic methods can now be paired with a slower chemogenetic modulation of a whole neuron population, enabling complex control of neuroactivity. Park et al. recently developed improved excitatory and inhibitor LMOs (LMO4 and iLMO4, respectively) and demonstrated their use in a rat model [148]. Specifically, unilateral destruction of dopaminergic fibers to the striatum was performed on rats to induce ipsilateral rotations after amphetamine stimulation. The authors then introduced an AAV vector with LMO4 into the right striatum and, after a few weeks, delivered CLZN or the vehicle control to the rats. After dosing with amphetamines, the number of ipsilateral turns was recorded. Strikingly, in mice with BRET-induced optogenetic stimulation, a nearly 3-fold drop in ipsilateral turns was recorded within the first 15 min compared to the controls, indicating a transient return of striatum dopaminergic drive. These results demonstrate a powerful way of temporally and spatially controlling neurological function and behavior without the use of invasive optic fibers for external light excitation by using BRET for photostimulation [156].

**Figure 13:**
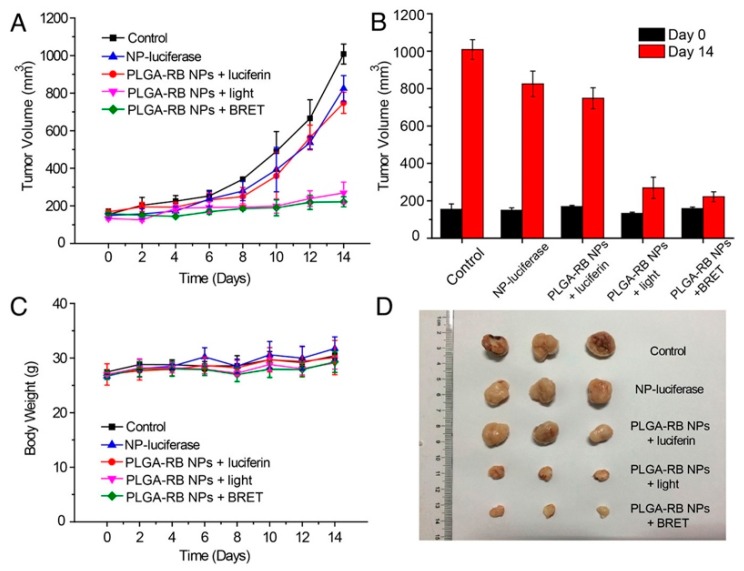
In vivo demonstration of BRET-mediated photodynamic therapy (PDT) using PLGA nanoparticles loaded with rose bengal (RB) and FLuc. (**A**) Tumor growth over time when treated with the PLGA NPs. Only the pink and green traces show data from animals dosed with all the elements necessary for PDT. The pink group was exposed to external excitation light as a more traditional approach to PDT, while BRET-mediated local excitation of the construct was used for PDT in the green group. (**B**) Tumor volume on day 0 and day 14 after PDT treatment. (**C**) Body weight change after intratumoral injections of the NPs showed no significant change, suggesting low toxicity of the PDT treatment. (**D**) Excised tumors from the five treated groups on day 14. Reprinted with permission from [157]. Copyright 2018, American Chemical Society.

Finally, BRET-based photoinduction has potential for therapeutic use as well, as demonstrated by Yang et al. [157]. The authors demonstrated efficient photodynamic therapy (PDT) using BRET-based localized excitation to inhibit tumor growth in a mouse model. This approach overcomes issues of excitation light penetration depth, skin photosensitivity, and photothermal injury. The PDT construct combined a biodegradable poly(lactic-co-glycolic acid) PLGA nanoparticle loaded with a reactive oxygen species (ROS)-generating photosensitizer, rose bengal (RB), bioconjugated to the BRET donor FLuc. In the presence of luciferin, BRET from FLuc to RB generates singlet oxygen, destroying cancer cells. Five groups of H22 tumor-bearing mice with tumor volumes of approximately 150 mm^3^ were injected intratumorally with PBS, PLGA-RB-FLuc nanoparticles, PLGA-RB nanoparticles plus luciferin, PLGA-RB nanoparticles (plus external light irradiation), or PLGA-RB-FLuc nanoparticles plus luciferin. As shown in Figure 13A, tumor growth was hindered by ROS generation for groups treated with PLGA-RB nanoparticles with external excitation (520 nm) and PLGA-RB-FLuc nanoparticles with luciferin. The control groups exhibited a 6-fold increase in tumor volumes on day 14 post-treatment (Figure 13B) with the last two PDT groups demonstrating substantial tumor growth inhibition. Low toxicity of the PDT treatment is supported by the comparable body weights for all groups (Figure 13C). Excised tumors from the five groups visually show the successful tumor growth inhibition by PDT for the PLGA-RB with external light excitation and PLGA-RB with BRET (Figure 13D). PDT using BRET is an interesting way to impact tumor cells in deep tissues, where external light sources struggle to penetrate [157].

## 5. In Vivo CRET

The subtle difference between BRET and CRET is based on whether the light-producing process evolved in nature for that purpose, such as luciferase, aequorin, and their lab-derived mutants, or if other chemical reactions, such as those utilizing redox enzymes or metal catalysts, are hijacked to produce photonic emission. In the section above, luciferase was bound to QDs to create BRET-based self-illuminating QDs. A similar approach for the CRET-based illumination of NIR-emitting QDs cleverly utilized the activity of endogenous enzyme to react with luminol as an in vivo sensor. Myeloperoxidase (MPO) is an enzyme that is highly active in neutrophils and macrophages and is present in sites of inflammation. MPO produces hypochloric acid, a potent oxidant that has been shown to induce chemiluminescence from luminol [158]. To enhance the tissue penetration of light produced by this reaction, Zhang et al. co-injected luminol and NIR-emitting QDs in mice exhibiting lung inflammation for CRET-based imaging [159]. Interestingly, even though the reactants were not conjugated to ensure their close proximity, as has been the case in the majority of our examples, the CRET signal was 37-fold brighter than luminol alone. Ex vivo fluorescence imaging demonstrated that the QDs were distributed throughout the body, but the CRET signal was exclusive to the inflamed lungs. The dependence of this signal on MPO activity was confirmed through several controls.

**Figure 14:**
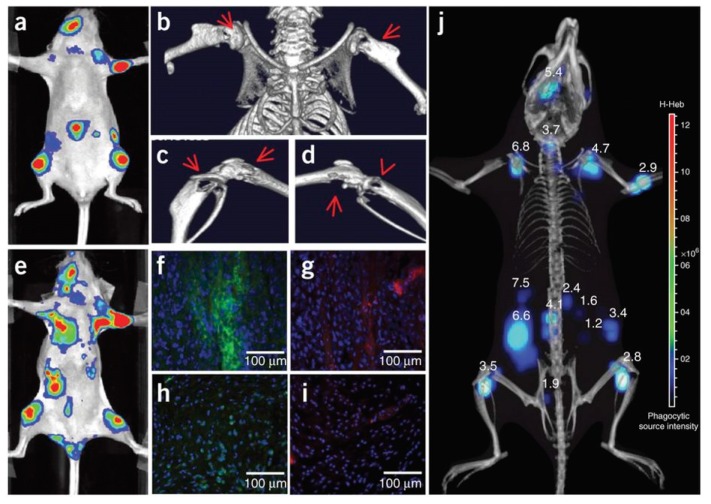
BLI, CRET and micro-computed tomography of the presence of MPO activity in MDA-MB-231-luc2 tumor metastases. (**a**) BLI of the tumor metastases 3 weeks after intracardiac injection of tumor cells. (**b**–**d**) Micro-CT imaging showing osteolytic lesions indicated by the red arrows at the metastasis tumor sites. (**e**) MPO activity in these lesions analyzed with Luminol-R. (**f**–**i**) Immunohistological analysis. (**f**,**g**) Tumor sites and (**g**,**i**) healthy tissues from a control mouse from a similar location as the metastases. (**f**,**h**) correspond to the staining of neutrophils and (**g**,**i**) to the staining of MPO. (**j**) Recording of 3-D CLI and micro-CT imaging showing the metastatic lesions in an anatomical configuration. Tumor depths measured with the reconstruction algorithm DLIT. Reprinted with permission from [159]. Copyright 2013, Springer Nature.

The authors used the same approach to image neutrophil invasion in deep tissue metastatic tumors [159]. Intracardiac injection of MDA-MB-231-luc2 tumor cells in nude mice induced tumor metastasis on the limbs, mandible, and inside the abdominal cavity after 3 weeks (Figure 14). Intravenous injection of Luminol-R (a mixture of luminol and NIR-emitting QDs) in the mice resulted in CRET signals due to MPO activity in the regions where the metastatic cells were present. Immunohistochemical analysis from the metastasized tumor and healthy tissues from a control mouse show increased staining for neutrophils and MPO from the tumor sites, confirming that the CRET mechanism is the same as seen in the inflammation case above. Micro-CT imaging and 3-D CLI (Figure 14j) showed the metastatic lesion in an anatomical configuration, and the tumor depths at different metastasis locations was measured using a reconstruction algorithm (DLIT). Tumor depths ranged from 1.2–6.8 mm, suggesting the efficacy of the CRET sensor for the monitoring of MPO in deep inflamed tissues [159].

**Figure 15:**
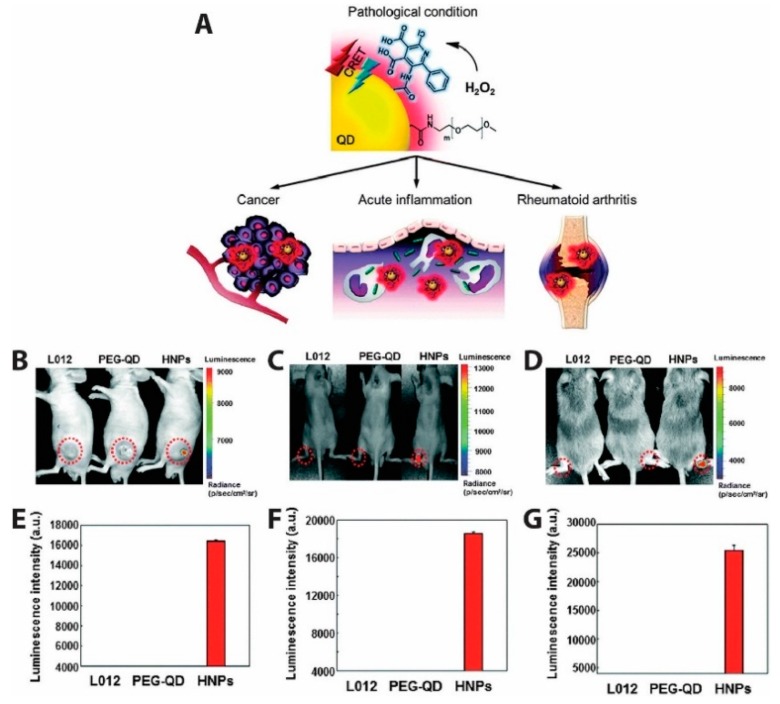
Hybrid nanoparticles (HNPs) with a conjugated luminol derivative (L012) produce NIR QD emission in response to reactive oxygen species in vivo. (**A**) Schematic representation of HNPs used for the detection of overproduced H_2_O_2_ in various diseases states. CL signals generated by CRET in presence of hydrogen peroxide in mice bearing (**B**) tumor, (**C**) acute inflammation, and (**D**) arthritis. CL signal was emitted from the HNPs in all three disease models, each of which involves the overproduction of H_2_O_2_. CL was not observed form either L012 or QDs alone. (**E**–**G**) Quantification of the CRET signal intensities. Higher CRET signals were obtained from the late stage inflammation model (**D**,**G**) than the early stage inflammation model (**C**,**F**), suggesting higher and long lasting ROS in the arthritis model. Adapted with permission from [161]. Copyright 2016, The Royal Society of Chemistry.

QD-based CRET was also used to measure the presence of endogenously produced hydrogen peroxide, a reactive oxygen species (ROS) believed to be an essential diagnostic marker as its overproduction is involved in the development of diseases such as cancer and arthritis [160]. Due to the low abundance and reactivity of H_2_O_2_ compared to other ROS molecules, it is challenging to image in vivo. This challenge was met by Lee et al., who developed hydrogen peroxide-responsive hybrid nanoparticles (HNPs) comprising PEG-coated QDs emitting at 800 nm and functionalized with a luminol derivative (L012) [161]. L012 is oxidized 100-fold more efficiently under physiologic conditions than traditional luminol, resulting in a substrate that is very sensitive to H_2_O_2_ concentration without requiring co-labeling with a chemiluminescent enzyme. In the presence of H_2_O_2_, CRET from L012 to the QDs produced NIR light that could be easily imaged (Figure 15). For in vivo chemiluminescence (CL) detection of H_2_O_2_ as depicted in Figure 15B–D, three models for diseases linked to hydrogen peroxide production (tumor, acute inflammation, and arthritis) were used. In the tumor model (Figure 15B), human prostate cancer cells (PC3) were injected into left flanks of mice and CL images acquired after intratumoral administration of L012, PEG-QD, or HNPs. Intraarticular injection of LPS injection produced the inflammation model (Figure 15C), while a collagen-induced arthritis (CIA, Figure 15D) in mice was used as a model for late stage inflammation. No CL was observed in normal mice injected with L012, PEG-QD, or HNPs, whereas the three disease model mice showed specific and high CL signals from HNPs. Quantified luminescent intensities from Figure 15F,G shows more H_2_O_2_ is present in late stage inflammation than in early stage inflammation. Specific signals emitted by HNPs in the presence of H_2_O_2_ demonstrated the ability of HNPs to detect irregular levels of hydrogen peroxide in the three disease models and shows promise as a diagnostic agent for H_2_O_2_ associated diseases [161].

**Figure 16:**
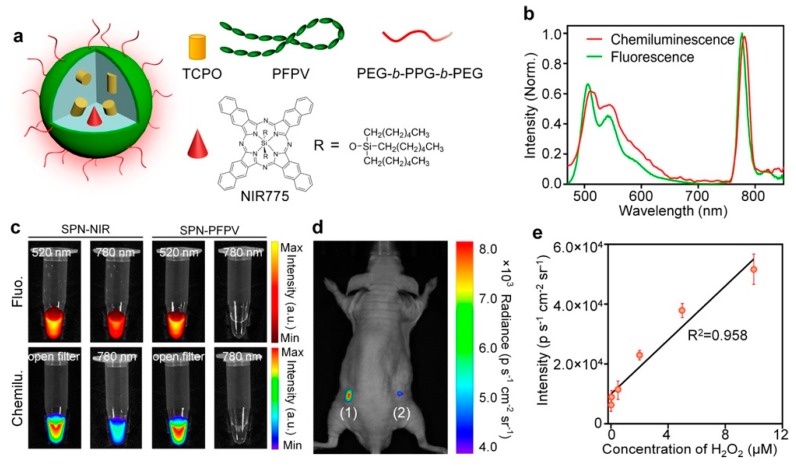
Semiconducting polymer nanoparticles emitting in the NIR (SPN-NIR) developed for CRET-based imaging of H_2_O_2_. (**a**) Schematic of the SPN-NIR. The polyfluorene-based semiconducting polymer PFPV, chemiluminescent substrate TCPO, and naphthalocyanine dye NIR775 were co-precipitated with the amphiphilic triblock copolymer PEG-*b*-PPG-*b*-PEG to create SPN-NIR particles with hydrodynamic diameters of 15–25 nm. (**b**) Chemiluminescence and fluorescence spectra of SPN-NIR. Chemiluminescence was induced by the addition of excessive H_2_O_2_ (10 mM). (**c**) Representative chemiluminescence and fluorescence images of SPNs (18 μg/mL, 0.1 mL) in the presence of H_2_O_2_ (10 mM). The fluorescence signals were detected at 520 or 780 nm; the chemiluminescence signals were detected with open filter or at 780 nm. (**d**) In vivo imaging of exogenous H_2_O_2_ using SPN-NIR. Representative chemiluminescence image of mouse with the subcutaneous implantation of (1) H_2_O_2_ (8 nM) + SPN-NIR (0.1 mg/mL, 0.1 mL) and (2) SPN-NIR (0.1 mg/mL, 0.1 mL). (**e**) In vivo chemiluminescence intensities of the subcutaneous inclusion of SPN-NIR as a function of the concentration of H_2_O_2_. Values are the mean ± s.d. for n = 3 mice. Reprinted with permission from [162]. Copyright 2016, American Chemical Society.

For the ultrasensitive imaging of H_2_O_2_ in a mouse model of peritonitis or neuroinflammation, Zhen et al. developed a chemiluminescent NIR reporter. They co-precipitated a luminescent semiconducting polymer, chemiluminescent substrate, and NIR-emitting dye with a polyethylene glycol (PEG)-based block-copolymer to generate doped semiconducting polymer nanoparticles (SPN) [162]. The polyfluorene-based semiconducting polymer (PFPV) is a luminescent reporter when paired with the substrate peroxalate bis(2,4,6-trichlorophenyl) oxalate (TCPO). As H_2_O_2_-dependent green chemiluminescence from this system would be rapidly attenuated by tissue scattering and absorption, a naphthalocyanine dye emitting around 775 nm (NIR775) was additionally incorporated into the SPNs, to create SPN-NIR (Figure 16). CRET from the green chemiluminescent reporter to NIR775 generates NIR emission in the presence of H_2_O_2_. To demonstrate this contrast agent in vivo, lipopolysaccharide (LPS), an endotoxin found on the surface of Gram-negative bacteria, was injected into mice intraperitoneally to induce peritonitis. Four hours later, SPN-NIR were intraperitoneally injected and CRET intensity acquired. The CRET images exhibit a 2.5-fold higher NIR signal for the LPS-treated mice than for the control saline mice and a 51% signal decrease in the presence of glutathione (GSH), which is an antioxidant and nucleophilic scavenger of ROS. In a separate experiment, H_2_O_2_ in the brain was measured following induced neuroinflammation induced by intracerebral injection of LPS. Since a maximum 5 µL volume can be injected into the mouse brain for safety reasons, only a 2 µL (10 mg/mL) of SPN-NIR could be administered in the inflamed neural tissue to detect H_2_O_2_. Even with the small quantity of SPN-NIR injected, an approximate 1.7-fold higher CL intensity was observed for LPS-treated mice than for the control mice and a reduction by 21% was observed in the presence of GSH. This study introduces the ability to used organic optical probes to sensitively image H_2_O_2_ in living animals without the need for external light excitation [162].

**Figure 17:**
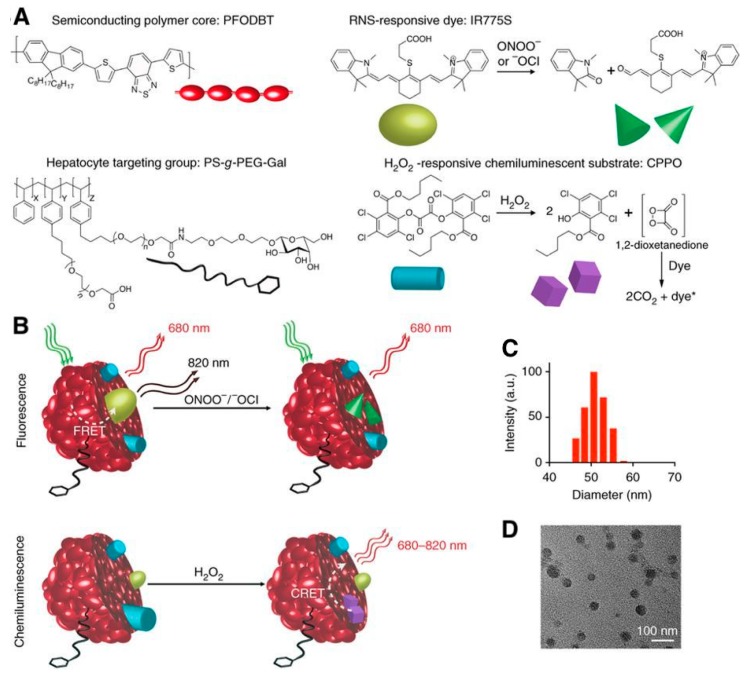
Combined CRET/FRET sensor for in vivo detection of drug-induced hepatotoxicity. (**A**) Molecular components of CF-SPN are the NIR fluorescent semiconducting polymer PFODBT, a PEG-grafted poly(styrene) copolymer conjugated to galactose for hepatocyte targeting (PS-g-PEG-Gal), the H_2_O_2_-specific chemiluminescent substrate CPPO that serves as CRET energy donor, and the FRET acceptor IR775S that degrades after oxidation by ONOO^−^ or ^−^OCl (dark green). PFODBT serves as the CRET energy acceptor and the FRET energy donor. (**B**) Illustration of the mechanism of simultaneous and differential detection of ONOO^−^ or ^−^OCl and H_2_O_2_ by CF-SPN. After drug challenge to the liver, CF-SPN report via the chemiluminescent and fluorescent channels the generation of radical metabolites at safe (**left**) and toxic (**right**) drug doses. (**C**) Hydrodynamic diameter distribution of CF-SPN, determined by dynamic light scattering. (**D**) Transmission electron micrograph of CF-SPNs. Adapted with permission from [163]. Copyright 2014, Springer Nature.

Shuhendler et al. used a complex SPN-dye combination to simultaneously sense H_2_O_2_, peroxynitrite (ONOO^−^), and hypochlorite (^−^OCl) in vivo in mice using both CRET and FRET in a single senor (Figure 17) [163]. The detection of both reactive oxygen and reactive nitrogen species is proposed as a screen for drug-induced hepatotoxicity, which has been associated with the production of these radical species. Two distinct sensors are combined in one particle, facilitating the simultaneous and differential detection of H_2_O_2_ and ONOO^−^/^−^OCl. CRET from the SPN proceeds similarly as described above: H_2_O_2_ reacts with a chemiluminescent substrate (a hydrophobic peroxyoxalate, bis-(2,4,5-trichloro-6-(pentyloxycarbonyl)phenyl)oxalate; CPPO) and excites the NIR fluorescent semiconducting polymer (poly(2,7-(9,9-dioctylfluorene)-*alt*-4,7-bis(thiophen-2-yl)benzo-2,1,3-thiadiazole); PFODBT) producing emission at 680 nm. In addition to being the CRET acceptor, PFODBT also acts as a FRET donor to the cyanine dye IR775S. Because IR776S is irreversibly degraded by ONOO^−^ and ^−^OCl, a decrease in the FRET-induced IR775S signal indicates the presence of these radicals. Incorporation of a galactosylated graft copolymer of poly(styrene) and poly(ethylene glycol) (PS-*g*-PEG-Gal) facilitates targeting of the particles to the liver, where it was used to probe for hepatotoxicity following dosing with acetaminophen or the anti-tuberculosis agent isoniazid. In vivo characterization of the sensor revealed specific targeting to the liver, no uptake dependency of CF-SPN upon drug treatment, and good stability with adequate imaging penetration depth. CF-SPN also did not produce any ROS and RNS when accumulated in the liver. This sensor successfully indicated the presence of reactive oxygen and nitrogen species in the livers of mice overdosed with acetaminophen (300 mg/kg/day) as well as the remediation of reactive species in response to antidote treatments with antioxidants.

## 6. Developing Technologies and Increased Interest

While in vivo energy transfer-based optical sensing remains a significant challenge for all the reasons detailed above, there are recent advancements in multiple fields that have the potential to increase both interest and application of in vivo RET-based sensing approaches. For example, although NIR-I light has far improved imaging depths compared to visible light, NIR-II and NIR-III emitters would enable even deeper tissue depths and better resolution than NIR-I. However, a lack of these short-wave infrared (SWIR) emitters has hindered the development of NIR-II or NIR-III RET sensors. Recently, Bawendi group presented the imaging capability of their SWIR core–shell and core–shell–shell QDs based on an indium arsenide core with emission between 900 to 1600 nm. They successfully functionalized the SWIR QDs and tested them in vivo in mice for contact-free cardiography and hemorrhage sensing, quantitative metabolic sensing, and mapping of the blood flow [164]. This demonstrates how imaging in the SWIR, which is between 1000–2000 nm, exploits the NIR-II and NIR-III biological windows to reduce scattering compared to imaging in the NIR-I. The development of a BRET-based SWIR emitting system would be an exciting addition to the field.

Tissue autofluorescence originating from excitation light in the UV/Vis often pollutes the detection channels of fluorescent probes in vivo, contributing to low signal-to-noise ratio of the recorded fluorescence [92,165,166]. Using a time domain (TD) method described by Kumar et al. for whole body small animal fluorescence imaging [167], Rice et al. showed that the TD method discriminated tissue autofluorescence from extrinsic fluorescence effectively, resulting in a 25-fold increase in sensitivity for fluorescence detection in subcutaneous tumors expressing GFP in nude mice [93]. Kumar et al. later showed that the TD method shows promise for the accurate identification of xenograft tumors in mice using indocyanine-green (ICG) [168]. The fluorescence lifetime of ICG bound to tumors could be differentiated from the fluorescence lifetime of tissue autofluorescence, hence providing a decent contrast of ICG fluorescence from tissue autofluorescence in the TD. By comparing TD with continuous wave methods, they demonstrated that fluorescence lifetime contrast resulted in more than 98% sensitivity and specificity and a 10-fold reduction in error rates compared to intensity based detection for ICG identification of tumors [168]. The TD fluorescence system developed for small animal whole body imaging could be applied to the monitoring of biological interactions using FRET while avoiding autofluorescence. Given that RET results in changes in luminescence lifetime for both the donor and acceptor fluorophores, TD fluorescence tomography could be used very effectively for in vivo RET-based sensing using lifetime rather than spectral wavelength or intensity as the output.

Finally, advances in bioluminescent systems are also increasing the probes available for sensor development. For example, interesting work identifying orthogonal luciferase–substrate pairs enables specific induction of multiple distinct bioluminescent emission peaks in a single organism. These substrate–enzyme pairs could be individually coupled with an effective NIR emitter to allow for multiplexed BRET sensing [169,170].

## 7. Conclusions

While FRET is more established and widely applied due to its extensive in vitro use, biosensing using BRET and CRET has significant advantages in vivo. A higher signal-to-noise ratio, deeper tissue imaging, and nominal autofluorescence compared to FRET demonstrate the advantage of eliminating the requirement for external excitation illumination. The addition of the bio/chemiluminescent substrate, however, adds an additional reagent to the reaction that must be delivered to the site of interest, adding a ‘drug delivery’ aspect to the experimental design. The most successful probes were NIR emitters that take advantage of the increased tissue penetration in the first optical tissue window. The emergence of novel ‘green’ fluorophores, such as cadmium-free, NIR-emitting QDs, and FDA approved dyes exploiting the NIR-I, NIR-II, and NIR-III optical windows providing increased penetration depth and spatial resolution hold promise for continued development of RET-based sensors for in vivo applications.

## Figures and Tables

**Table 1:** Examples of BL and CL reporters used in BRET and CRET applications [20,22,28,29,30]

**BRET**			
**Reporter Genes (Luciferases, Photoproteins)**	**Luciferin (Substrate)**	**BL Emission Max. (nm)**	**Required Components (Oxidant; Cofactors)**
FLuc	D-luciferin	557	O_2_; ATP, Mg^2+^
RLuc	Coelenterazine DeepBlueC	480 395	O_2_ O_2_
GLuc	Coelenterazine	480	O_2_
NLuc	Fumarazine Fumarazine28 Fumarazine30	460 568 598	O_2_ O_2_ O_2_
Aeq	Coelenterazine	469	Ca^2+^
Obe	Coelenterazine	485	Ca^2+^
**CRET**			
**Catalyst**	**Substrate**	**CL emission max. (nm)**	**Required components (oxidant; cofactors)**
Fe^2+^	Luminol	455	H_2_O_2_
HRP	Luminol	425	H_2_O_2_; OH^−^
HRP	Acridan	530	H_2_O_2_
Alkaline phosphatase	Adamantyl,2-dioxetane	480, 530	

FLuc: firefly luciferase; RLuc: Renilla luciferase; GLuc: Gaussia Luciferase; NLuc: NanoLuc™ luciferase; Aeq: aequorin; Obe: obelin; HRP: horseradish peroxidase.
